# Behavioral Treatment for Speech and Language in Primary Progressive Aphasia and Primary Progressive Apraxia of Speech: A Systematic Review

**DOI:** 10.1007/s11065-023-09607-1

**Published:** 2023-10-04

**Authors:** Lisa D. Wauters, Karen Croot, Heather R. Dial, Joseph R. Duffy, Stephanie M. Grasso, Esther Kim, Kristin Schaffer Mendez, Kirrie J. Ballard, Heather M. Clark, Leeah Kohley, Laura L. Murray, Emily J. Rogalski, Mathieu Figeys, Lisa Milman, Maya L. Henry

**Affiliations:** 1 Department of Speech, Language, and Hearing Sciences, University of Texas at Austin, 2504A Whitis Ave. (A1100), 78712 Austin, TX USA; 2 School of Psychology, University of Sydney, 2006 Sydney, NSW Australia; 3 Department of Communication Sciences and Disorders, University of Houston, Houston, TX 77204 USA; 4 Department of Neurology, Division of Speech Pathology, Mayo Clinic, Rochester, MN 55902 USA; 5 US Department of Communication Sciences and Disorders, Faculty of Rehabilitation Medicine, University of Alberta, T6G 2R3 Edmonton, AB Canada; 6 The University of St. Augustine, Austin, TX 78712 USA; 7 Faculty of Medicine & Health and Brain & Mind Centre, University of Sydney, Sydney, NSW 2006 Australia; 8 School of Communication Sciences and Disorders, Western University, London, ON N6A 3K7 Canada; 9 Department of Psychiatry and Behavioral Sciences, Northwestern University, Feinberg School of Medicine, 60611 Chicago, IL USA; 10 Mesulam Center for Cognitive Neurology and Alzheimer’s Disease, Northwestern University, Feinberg School of Medicine, 60611 Chicago, IL USA; 11 Faculty of Rehabilitation Medicine, University of Alberta, Edmonton, AB T6G 2R3 Canada; 12 Department of Communicative Disorders and Deaf Education, Utah State University, Logan, UT 84322 USA; 13 Department of Neurology, Dell Medical School, University of Texas at Austin, 78712 Austin, TX USA

**Keywords:** Primary progressive aphasia, Primary progressive apraxia of speech, Semantic dementia, Frontotemporal dementia, Speech therapy, Systematic review

## Abstract

Primary progressive aphasia (PPA) and primary progressive apraxia of speech (PPAOS) are neurodegenerative syndromes characterized by progressive decline in language or speech. There is a growing number of studies investigating speech-language interventions for PPA/PPAOS. An updated systematic evaluation of the treatment evidence is warranted to inform best clinical practice and guide future treatment research. We systematically reviewed the evidence for behavioral treatment for speech and language in this population. Reviewed articles were published in peer-reviewed journals through 31 May 2021. We evaluated level of evidence, reporting quality, and risk of bias using a modified version of the American Speech-Language Hearing Association (ASHA) Levels of Evidence, an appraisal point system, additional reporting quality and internal/external validity items, and, as appropriate, the *Single Case Experimental Design* *Scale* or the *Physiotherapy Evidence Database – PsycBITE* *Rating Scale for Randomized and Non-Randomized Controlled Trials*. Results were synthesized using quantitative summaries and narrative review. A total of 103 studies reported treatment outcomes for 626 individuals with PPA; no studies used the diagnostic label PPAOS. Most studies evaluated interventions for word retrieval. The highest-quality evidence was provided by 45 experimental and quasi-experimental studies (16 controlled group studies, 29 single-subject designs). All (*k* = 45/45) reported improvement on a primary outcome measure; most reported generalization (*k* = 34/43), maintenance (*k* = 34/39), or social validity (*k* = 17/19) of treatment for at least one participant. The available evidence supports speech-language intervention for persons with PPA; however, treatment for PPAOS awaits systematic investigation. Implications and limitations of the evidence and the review are discussed.

**Supplementary Information:**

The online version contains supplementary material available at 10.1007/s11065-023-09607-1.

## Introduction

Primary progressive aphasia (PPA) is a debilitating neurodegenerative syndrome in which progressive deterioration of language (with or without motor speech impairment) presents as the prominent symptom (Gorno-Tempini et al., 2011; Goyal et al., 2021; Vitali, 2022) with relative sparing of other cognitive, motoric, and behavioral functions (Mesulam, 1982, 2001; Montembeault et al., 2018; Sonty et al., 2003; Tee & Gorno-Tempini, 2019). A related disorder that has been defined more recently is primary progressive apraxia of speech (PPAOS), which affects speech praxis in the absence of significant linguistic deficits (Duffy et al., 2015). PPA and PPAOS are now identified with greater accuracy and frequency, and individuals with PPA and PPAOS, as well as their families, increasingly seek options for treatment. With distinctive language or speech features and an inevitable decline in function, these syndromes present unique clinical challenges for speech-language clinicians.

There are no pharmacologic agents proven to significantly benefit or halt the progression of the core speech/language symptoms in PPA/PPAOS (Tsai & Boxer, 2016). Modest effects of pharmacological treatment for Alzheimer’s disease may improve function in some persons with logopenic PPA or other PPA/PPAOS syndromes associated with Alzheimer pathology (Kobayashi et al., 2016; Trinh et al., 2003), and some selective serotonin reuptake inhibitors (SSRIs) can improve behavior in PPA syndromes associated with frontotemporal lobar degeneration (Ljubenkov & Boxer, 2021). Historically, however, persons with PPA/PPAOS have been offered few non-pharmacologic treatments for speech, language, and communication, despite the recent proliferation of empirical studies investigating such interventions in this population. This has been due both to limited understanding of PPA and PPAOS by health professionals, and to uncertainty regarding the value, feasibility, and long-term benefit of behavioral treatment for individuals facing progressive disease. There is, therefore, a critical need for evidence addressing the efficacy of interventions to improve or maintain speech and language in PPA and PPAOS. A new systematic evaluation of the treatment evidence is warranted to inform best clinical practice and guide future treatment research. The present systematic review focuses on behavioral (i.e., non-pharmacological, non-neuromodulatory) treatments for speech and language symptoms associated with PPA and PPAOS. As background to the review, we describe the current diagnostic framework for PPA and PPAOS, the impacts of these disorders, the behavioral interventions available for speech and language in PPA, and the rationale and objectives for the systematic review.

### Current Diagnostic Framework for PPA and PPAOS

Current diagnostic criteria (Gorno-Tempini et al., 2011; Mesulam, 2001) for PPA require that a progressive communication deficit is the most prominent clinical feature and primary cause of impaired activities of daily living. The communication impairment must be caused by neurodegenerative disease and not by other neurological or psychiatric conditions. Prominent non-language impairments (cognitive, motoric, or behavioral disturbance) early in the disease process are exclusionary.

Three clinical variants of PPA are widely recognized by clinical and research communities. The nonfluent/agrammatic variant (nfvPPA) is characterized by agrammatic language production (i.e., utterances that lack appropriate grammatical structure) or effortful, halting speech (motor speech impairment consistent with apraxia of speech with or without dysarthria; Gorno-Tempini et al., 2011; Rohrer et al., 2010a, b). At least two of the following associated features must be present: impaired syntax comprehension, spared single word comprehension, and intact object knowledge. Neuroimaging shows left posterior-frontal/insular atrophy (Mandelli et al., 2018). The semantic variant (svPPA) presents with impaired confrontation naming (i.e., difficulty naming an object or its picture) and single word comprehension, with at least three of the following associated features: impaired object knowledge, surface dyslexia or dysgraphia (i.e., difficulty reading or spelling irregular words [e.g., “colonel”]), spared repetition, spared grammar, and spared motor speech abilities (Gorno-Tempini et al., 2011; Hodges & Patterson, 2007). Neuroimaging shows a characteristic (left hemisphere greater than right) anterior temporal pattern of atrophy (Hodges et al., 1992; Rosen et al., 2002). The logopenic variant (lvPPA) is characterized by impaired word retrieval and phrase/sentence repetition, as well as three of the following associated features: phonological errors (i.e., well-articulated sound errors) in speech production, spared grammar, spared single word comprehension, and spared motor speech abilities (Conca et al., 2022; Gorno-Tempini et al., 2011; Rohrer et al., 2010a, b). Atrophic changes are predominantly observed in left temporoparietal areas (Gorno-Tempini & Brambati, 2008; Teichmann et al., 2013). Whereas the three variants specified by the consensus paper account for the majority of cases, unclassifiable and “mixed” PPA presentations comprise 10–41% of reported cases (Gil-Navarro et al., 2013; Gorno-Tempini et al., 2011; Harris et al., 2013; Mesulam et al., 2012; Sajjadi et al., 2012; Utianski et al., 2019). Histopathological findings overlap across the three consensus variants and include FTLD-tau inclusions, transactive response DNA-binding protein of 43kDA (TDP-43) inclusions, and Alzheimer-type pathology, with nfvPPA most likely to be associated with FTLD tau, svPPA with TDP-43, and lvPPA with Alzheimer pathology (Gorno-Tempini et al., 2011; Rohrer et al., 2012; Spinelli et al., 2017).

Individuals with PPA eventually develop additional cognitive, behavioral, and motor features. Those with nfvPPA may experience executive function deficits and motor symptoms with progression (Karageorgiou & Miller, 2014; Kertesz et al., 2005); those with svPPA may develop face processing deficits, disrupted sleep, changes in appetite, changes in libido, emotional blunting, disinhibition, and other changes in behavior (Karageorgiou & Miller, 2014); those with lvPPA may develop episodic memory deficits (Eikelboom et al., 2018; Leyton et al., 2013; but for an alternative view see Mesulam et al., 2021), limb apraxia, and visuospatial deficits (Marshall et al., 2018).

The diagnosis of PPAOS has been applied (Duffy et al., 2015; Josephs et al., 2012) in cases where progressive apraxia of speech (AOS) occurs in the absence of significant language decline. There are changes in speech timing (e.g., slow speech rate, slow transitions between speech sounds within words, pauses between syllables, and lengthened speech sounds) and reduced accuracy in pronouncing speech sounds (e.g., distortions, additions, repetitions, and substitutions of sounds), attributed to disordered planning or programming of sensorimotor commands for speech movements (Botha & Josephs, 2019). Histopathology in well-characterized cases is almost exclusively FTLD-tau (Botha & Josephs, 2019), and in a longitudinal case series, five of 13 individuals with PPAOS developed the motoric and other deficits seen in progressive supranuclear palsy syndrome (Josephs et al., 2014).

In the absence of population-wide epidemiological studies, it is difficult to reliably estimate the number of individuals with PPA or PPAOS (see Supplementary Materials 1 for details about the following estimates). Existing reports have yielded estimates of PPA prevalence in the order of 3–4 per 100,000 (3.66 per 100,000 estimated from Coyle-Gilchrist et al. (2016); 3.23 per 100,000 reported by Magnin et al. (2016); 3.54 per 100,000 estimated from Borroni et al. (2010)). There have been no systematic epidemiological studies of PPAOS, but one estimate suggested that the prevalence of PPAOS may be close to 2 per 100,000 (Botha & Utianski, 2020).

### Impacts of PPA and PPAOS on the Individual, Their Close Others, and Community

The lived experience of a progressive language or speech disorder differs across individuals due to variability in disease factors (symptoms, severity, and rate of decline) as well as unique personal and social factors. The impacts include direct consequences of linguistic, motoric, and cognitive changes, including difficulty with communicating, completing activities of daily living (Moeller et al., 2021; O’Connor et al., 2014; Wicklund et al., 2007), and participating in life roles such as parenting, working, and maintaining social relationships (Morhardt et al., 2015, 2019; Weintraub et al., 2005). There are also legal and financial consequences (Kindell et al., 2015), which may be magnified for individuals with symptom onset prior to 65 years (Karageorgiou & Miller, 2014), as they are still in their prime earning years (Galvin et al., 2017) and may have significant financial, occupational, and parenting responsibilities.

There are also psychoemotional impacts for individuals with PPA and their close others (Cuijpers, 2005; Davies & Howe, 2019; Duffy et al., 2020; Kindell et al., 2014; Medina & Weintraub, 2007; Ruggero et al., 2019) such as feelings of embarrassment, self-consciousness, and frustration over communication difficulties, or worry, anxiety, and grief associated with the prognosis of progressive decline. Additionally, PPA care partners find communication increasingly difficult with disease progression (Davies & Howe, 2019; Riedl et al., 2014) and may grieve the loss of emotional connection with their loved one (Kindell et al., 2014; Pozzebon et al., 2016). It is unsurprising, therefore, that depression is common in people with PPA and their significant others (Cuijpers, 2005; Medina & Weintraub, 2007; Ruggero et al., 2019).

### Interventions for PPA and PPAOS

The devastating and complex effects of PPA and PPAOS have motivated a wide range of clinical intervention services and treatment research. In the absence of proven pharmacological agents, behavioral treatment has the greatest potential to ameliorate the impacts of speech, language, and communication impairments in PPA (Hodges & Piguet, 2018; Rogalski & Khayum, 2018). Behavioral treatment may target speech, language, and communication using listening, speaking, reading, writing, and other communicative activities and tasks. Targeted behaviors include but are not limited to receptive language processing and comprehension, intelligible speech production, lexical retrieval (i.e., word finding), sentence or discourse production, use of communication devices or other alternative communication modalities, and strategies for conversation and functional communication.

Four previous systematic reviews (Cadório et al., 2017; Carthery-Goulart et al., 2013; Cotelli et al., 2020; Volkmer et al., 2020a) have investigated treatment outcomes from behavioral interventions for communication in PPA. These reviews evaluated the evidence base supporting recommendations for cognitive rehabilitation (Carthery-Goulart et al., 2013), treatment maintenance and generalization following semantic therapy (Cadório et al., 2017), efficacy of lexical retrieval treatment with and without accompanying transcranial direct current stimulation (tDCS) (Cotelli et al., 2020), and the effectiveness and key components of functional communication interventions (Volkmer et al., 2020a); see Supplementary Materials 2 for additional information. These reviews concluded that there is sufficient evidence to recommend lexical retrieval treatment as a “Practice Option” (Cicerone et al., 2000) for individuals with svPPA (Carthery-Goulart et al., 2013); that there is some maintenance of direct lexical retrieval treatment gains at varying intervals post-treatment for many individuals (Cadório et al., 2017); that augmenting lexical retrieval treatment with tDCS results in small, direct, and generalized gains as well as maintenance of those gains (Cotelli et al., 2020); and that activity- and participation-directed communication interventions can lead to gains in functional communication (Volkmer et al., 2020a).

### Rationale for Systematic Review

Despite a robust literature base documenting the benefits of speech-language interventions for aphasia (Brady et al., 2016; Breitenstein et al., 2017; Shrubsole et al., 2016) and apraxia of speech (Ballard et al., 2015; Munasinghe et al., 2023; Wambaugh et al., 2006) caused by stroke, treatment for speech or language disorders in PPA and PPAOS is less widely implemented and evaluated. There are low referral rates (Taylor et al., 2009; Volkmer et al., 2020b) and continued clinical skepticism among referring and treating clinicians regarding the potential benefit of intervention for progressive disorders, especially regarding communicative impact beyond the clinic (i.e., social validity; Cupit et al., 2010).

The current review was undertaken as new behavioral treatment studies have proliferated since the first systematic review investigated primary outcomes, generalization, and maintenance of cognitive rehabilitation outcomes in PPA (Carthery-Goulart et al., 2013), because the social validity of behavioral speech or language treatment for PPA has not yet been systematically reviewed, and because no systematic review of treatment for PPAOS has been conducted.

### Objectives

The Academy of Neurologic Communication Disorders and Sciences (ANCDS) convened a panel of experts in PPA and PPAOS to conduct a systematic review of studies reporting treatment outcomes from behavioral (non-pharmacologic, non-neuromodulatory) interventions targeting speech or language in PPA or PPAOS. Studies of behavioral treatments administered in conjunction with pharmacological and neuromodulatory treatments were also included. The aim of the review was to address the following questions:

1. What evidence exists to support behavioral treatment for speech or language in PPA and PPAOS, and what is the quality and credibility of this evidence?
2. Does the evidence indicate that behavioral treatment for speech or language in PPA or PPAOS results in (i) improvement in trained skills/targets compared to baseline, control condition, or a comparison group; (ii) improvement in untrained skills/targets (generalization); (iii) maintenance of treatment gains beyond the immediate post-treatment period; or (iv) socially validated treatment gains?

## Methods

The procedure for this review was adapted from protocols developed by the ANCDS systematic review committee for AOS (Ballard et al., 2015; Wambaugh et al., 2006) and followed PRISMA reporting guidelines (Liberati et al., 2009). The ANCDS Evidence-Based Clinical Research Committee Chair appointed the review committee chair (MH), who then invited ANCDS members and others to participate in the review. The committee comprised 12 individuals from the USA, Canada, and Australia with extensive research and clinical experience with PPA or PPAOS and two additional members who assisted with procedural aspects of the review.

### Eligibility Criteria

For inclusion in the review, studies were required to (a) be original research (i.e., studies reporting on new research conducted by the study authors themselves); (b) be published before May 31, 2021, in a peer-reviewed journal (exclusive of conference abstracts); (c) investigate behavioral treatment for speech or language; (d) be conducted with one or more persons with a diagnosis of PPA or PPAOS; and (e) report treatment outcomes for at least one individual. Studies of behavioral treatment administered in conjunction with neuromodulation or sham neuromodulation, or in conjunction with a pharmacological agent or placebo, were included, but studies in which tDCS or a pharmacological agent were administered in the absence of behavioral treatment or in conjunction with a non-therapeutic task were not included.

### Information Sources

The following nine databases were queried: Medline; CINAHL (all via EBSCOhost); Embase; PsycInfo; ComDisDome; Scopus; and Web of Science. In consultation with a research librarian, an initial search was conducted on August 22, 2016, and was updated on October 6, 2020, and June 9, 2021.

### Search Strategy

The following search terms using Boolean operators were applied in all databases: (“primary progressive aphasia” or “primary progressive apraxia” or “semantic dementia”) AND TS = ( tdcs OR “transcranial direct current stimulation” OR (( semantic OR speech OR behavior* OR behaviour* OR language OR spelling OR naming OR anomia) NEAR/5 (therap* OR treatment OR intervention* OR train* OR rehab*)) OR “speech language”). TMS and transcranial magnetic stimulation were not included as search terms; the small number of TMS treatment studies identified via hand-searching did not include a behavioral treatment component. The search was limited to papers written in the English language.

Reference lists from previously published reviews and from papers included in the review were hand-checked for additional relevant titles and the abstracts of those articles were also screened.

### Study Selection

Covidence software was used for screening of studies. The software automatically removed duplicates at the time articles were imported. Two reviewers then independently screened titles and abstracts for relevance and adherence to inclusion criteria. Next, two reviewers independently screened full texts of the remaining articles following the title/abstract screening to ensure they met inclusion criteria for formal review and contained sufficient detail for analysis. The proportion of studies with inter-rater agreement regarding eligibility for inclusion was 0.97 for the title/abstract screen and 0.99 for the full text screen. Disagreements between reviewers were resolved via consensus. Excluded articles were not evaluated further.

### Data Collection

Articles meeting inclusion criteria were distributed among the review committee members, and data from each article were extracted independently by two raters. The data extraction table and manual were modeled on materials used in previous ANCDS systematic reviews (Ballard et al., 2015; Wambaugh et al., 2006) and adapted based on participant, treatment, and study design characteristics relevant for PPA/PPAOS behavioral treatment studies. Raters were not assigned papers on which they were an author or had any involvement in conducting the research. Inconsistencies between raters were resolved via consensus by a subset of committee members. Subsequently, two authors (LW and MH) reviewed consensus ratings for study type, reporting quality, risk of bias, and level of evidence for treatment efficacy for all studies to ensure consistency across all rater pairs.

### Data Items

The following data items were extracted from studies meeting inclusion criteria. The completed data extraction table showing the consensus data is available at 10.17605/OSF.IO/Z5U8D (Henry et al., 2023).

#### Participant Characteristics and Diagnostic Information

The number of individuals with PPA or PPAOS who underwent behavioral treatment for speech or language and the number of control/other individuals were extracted. Individuals with PPA or PPAOS were assigned to the following subtypes according to the diagnosis given by original study authors: nfvPPA (including progressive nonfluent aphasia, or PNFA, which was considered synonymous with nfvPPA for the purpose of this review; PNFA was used as the diagnostic label in only two studies included in the review), svPPA (including semantic dementia, which was considered synonymous with svPPA for the purpose of this review), lvPPA, mixed PPA, PPA not otherwise specified, and PPAOS^7^.

Reviewers assigned a rating of 1–5 for each study to characterize the level of evidence provided for the diagnosis of PPA, with level 1 indicating that the diagnosis for PPA and subtype were well supported by descriptive information and assessment data, level 2 indicating that the diagnosis of PPA but not the subtype was well supported, level 3 indicating inadequate information to confirm PPA diagnosis or subtype but with no exclusionary features noted, level 4 indicating no description of characteristics in support of the diagnosis, and level 5 indicating descriptions that were contradictory to the stated criteria for the diagnosis of PPA or subtype or that no diagnostic criteria were indicated. A table describing the levels of evidence for diagnosis of PPA in more detail is provided in Supplementary Materials 3. A similar 1–5 rating scheme was used to characterize the diagnostic evidence for participants who were stated to exhibit AOS, adapted from previous reviews (Ballard et al., 2015; Wambaugh et al., 2006). A description of the corresponding levels of evidence for diagnosis of AOS is provided in Supplementary Materials 4.

To further characterize participants in each study, the following information was extracted, when available: age, sex, time post-onset of symptoms, as well as sociodemographic, clinical history, speech, language, cognitive, and behavioral characteristics.

#### Treatment Characteristics

Raters briefly described treatment targets (the behaviors that treatments are intended to change; Fridriksson et al., 2022) and treatment techniques (activities or approaches used to effect learning and to improve communication skills). They also extracted mode of treatment delivery (e.g., in-person, telerehabilitation); number, frequency, and duration of treatment sessions; whether/what type of independent practice (e.g., homework) was conducted; and whether treatment fidelity data were reported.

#### Effects of Treatment

For each paper, raters described the primary outcome measure and any secondary outcome measures. To characterize treatment outcomes, raters noted whether the authors reported a significant change in the primary outcome measure(s) compared to baseline or a control or comparison group or control items. Raters also documented, when reported, generalization of treatment effects to untrained behaviors, untrained stimuli, or different contexts/settings; maintenance of outcomes and time-point(s) at which maintenance was measured; and evidence of social validity. Raters noted whether maintenance was measured relative to treatment onset or relative to the post-treatment phase. Outside the scope of the current review, raters also made observations about candidacy for treatments, authors’ rationales for treatments, and authors’ interpretations of study findings; these can be found at 10.17605/OSF.IO/Z5U8D (Henry et al., 2023).

### Critical Appraisal

#### Study Types and Levels of Evidence for Treatment Efficacy

Study design type (e.g., single-case experimental design, group experimental study) was extracted independently by two raters and each study was assigned a level of evidence following a modified version of the American Speech and Hearing Association (ASHA) evidence classification scheme for studies of treatment efficacy (ASHA, 2004) (with additional review for consensus and consistency within and across rater pairs, as noted above). The ASHA scheme is itself adapted from the Scottish Intercollegiate Guideline Network (SIGN, www.sign.ac.uk; Harbour & Miller, 2001) Levels of Evidence framework; see Fig. 2 for further details. The ASHA Levels of Evidence for Studies of Treatment Efficacy are ranked according to quality and credibility from highest/most credible (level Ia) to lowest/least credible (level IV). The levels of evidence for studies of treatment efficacy in this review were defined as follows:

- *Level Ia*—well-designed meta-analyses of > 1 randomized controlled trial (RCT)
- *Level Ib*—well-designed randomized controlled study (where “controlled” means inclusion of a comparison group who were untreated or underwent a different treatment condition; “randomized” means that participants were randomly assigned to treatment conditions)
- *Level IIa*—well-designed controlled study without randomization (where “controlled” means inclusion of a comparison group who were untreated or underwent a different treatment condition)
- *Level IIb*—well-designed quasi-experimental studies (i.e., within-group pre-post studies with control condition/items, Single-Case Experimental Design (SCED) studies as defined by Tate et al. (2008, 2013), and single-subject pre-post designs that met the following additional criteria (hereafter designated as SSPP +): provided at least two measurements of the target behavior in the baseline phase, a statistical analysis of change across study phases that accounted for the two baseline measurements, and a control condition or control items
- *Level III*—well-designed nonexperimental studies (i.e., correlational and case studies, case series, group studies lacking a control group/condition/items) and single-subject pre-post designs (SSPP) not meeting the criteria for SSPP + 
- *Level IV*—expert committee report, consensus conference, clinical experience of respected authorities

Level of evidence was determined based on the experimental conditions/study design used to evaluate the effect of behavioral treatment, not the design of conditions evaluating pharmacological or neuromodulatory intervention where these were also administered. For example, in neuromodulation studies where participants were randomized to neuromodulation and sham groups to evaluate the effect of neuromodulation on treatment outcomes (providing level 1b evidence for the effect of neuromodulation), but where the overall effect of the behavioral intervention was only evaluated via within-group comparisons with control items (e.g., Cotelli et al., 2014, 2016), the study was rated level IIb.

#### Appraisal Point System

All studies were rated for reporting quality and risk of bias using an appraisal point system (hereafter, APS), which addresses study quality in both single-subject and group designs (Oren et al., 2014). The APS contains nine items, five of which are double-weighted, receiving two points each (adequate comparison/control condition, random assignment of participants to treatment condition, adequate statistical reporting, clear reporting of materials, clear reporting of procedures), and four of which are single-weighted, receiving one point each (rationale for the study, participant description, clinical significance, and discussion/interpretation). The maximum score is 14 points.

#### Single-Case Experimental Design Scale

SCED studies (i.e., withdrawal/reversal, multiple-baseline, alternating treatments, and changing-criterion designs) were rated using the Single-Case Experimental Design scale (Tate et al., 2008, 2013) in addition to the APS.

#### Physiotherapy Evidence Database—PsycBITE Scale

Controlled group studies were rated using the Physiotherapy Evidence Database—PsycBITE (PEDro-P) scale (Murray et al., 2013), suitable for evaluating the methodological quality of RCT and non-randomized controlled trials. Studies were only rated on PEDro-P if the controlled design is applied to behavioral treatment*.*

#### Additional Characteristics

Several additional characteristics related to study reporting and internal/external validity were also extracted: replicability of study procedures, reliability of outcome measures, and replication of treatment effects within a study.

### Summary Measures

Participant information, including PPA subtype, age, sex, and time post-onset as well as information regarding treatment targets was summarized as counts for categorical variables, values for numerical variables in single-subject designs, or means with standard deviations for numerical variables in group studies. The number of studies that employed each study design type and the number of studies providing each level of evidence were calculated. The number of positive ratings for each item on the APS and SCED scales were summed across studies to calculate the proportion of studies that received credit for each individual scale item. Inter-rater agreement on the itemized ratings for APS, SCED, and PEDro-P prior to the resolution of rater discrepancies was calculated.

Treatment characteristics and outcomes data from the level I and level II studies that received 7 or more points on the APS were summarized separately. This criterion was determined a priori to select the higher-quality studies among those meeting the inclusion criteria for the systematic review. Following Oren and colleagues (Oren et al., 2014), we considered a score of 7 or greater on the APS to indicate adequate reporting and acceptable risk of bias. To score a minimum of 7 points on the APS, a study had to receive full points on at least two of the five double-weighted items, which are most important for internal validity. Regarding treatment outcomes, an outcome was operationally defined as positive when there was a significant change on a primary outcome measure in the desired direction, stability relative to a condition in which decline was observed, or when generalization, maintenance, or social validation was observed, based on the reported results of the data analysis procedures selected by study authors. For group studies, outcomes were determined at the group level. For studies that included a series of single subject designs, a study was considered to show a positive outcome if at least one of the participants met the criteria noted above.

### Synthesis of Results

Results were compiled using quantitative summaries and narrative synthesis.

## Results

### Study Selection

**Fig. 1:**
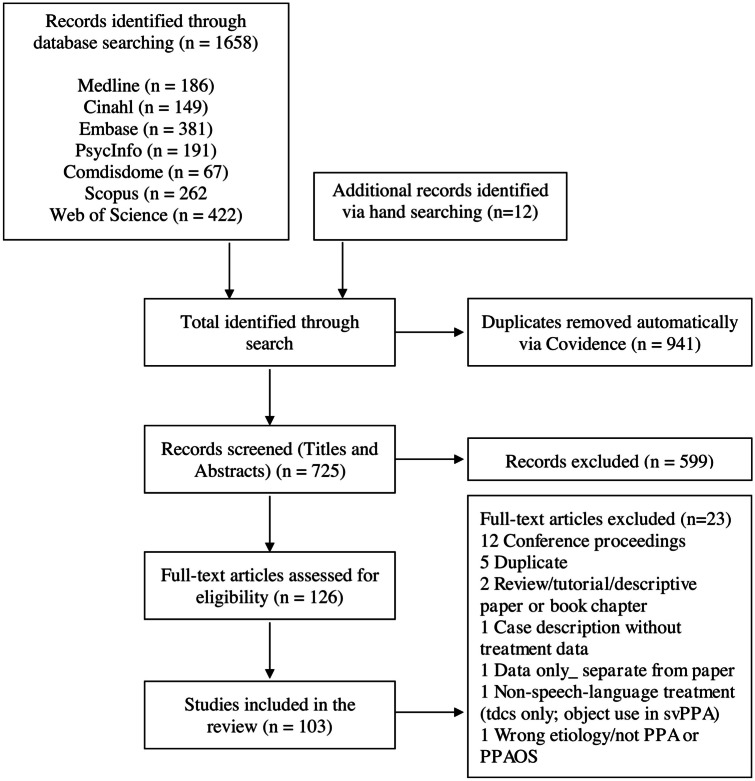
Flow diagram illustrating search process

A total of 103 studies reporting behavioral treatment for speech or language in PPA between 1994 and May 31, 2021, were identified for inclusion (see Supplementary Materials 5). The flow diagram for the search is shown in Fig. 1. Database searching, augmented by hand-searching citations within included studies, produced a total of 1670 citations, with 725 remaining after automatic removal of duplicates. Of these, 599 were discarded after reviewing the titles and abstracts because they did not meet the search criteria. The full text of the remaining 126 citations was examined and an additional 23 papers were discarded because they were not original peer-reviewed research articles or did not report behavioral treatment for speech or language in one or more individuals with PPA or PPAOS.

### Study Characteristics

Data extracted for the 103 studies are shown in Table 1.

**Table 1:**
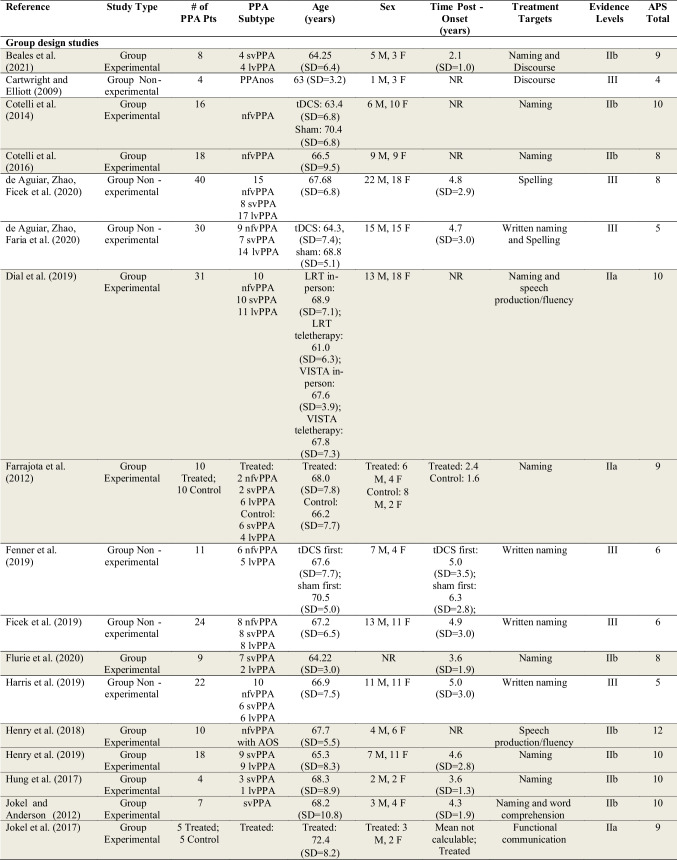

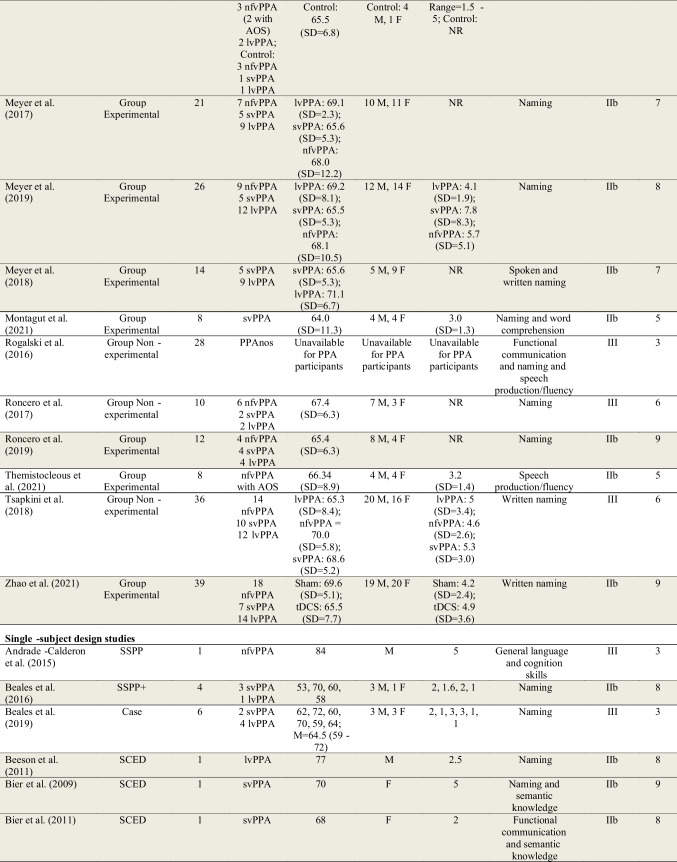

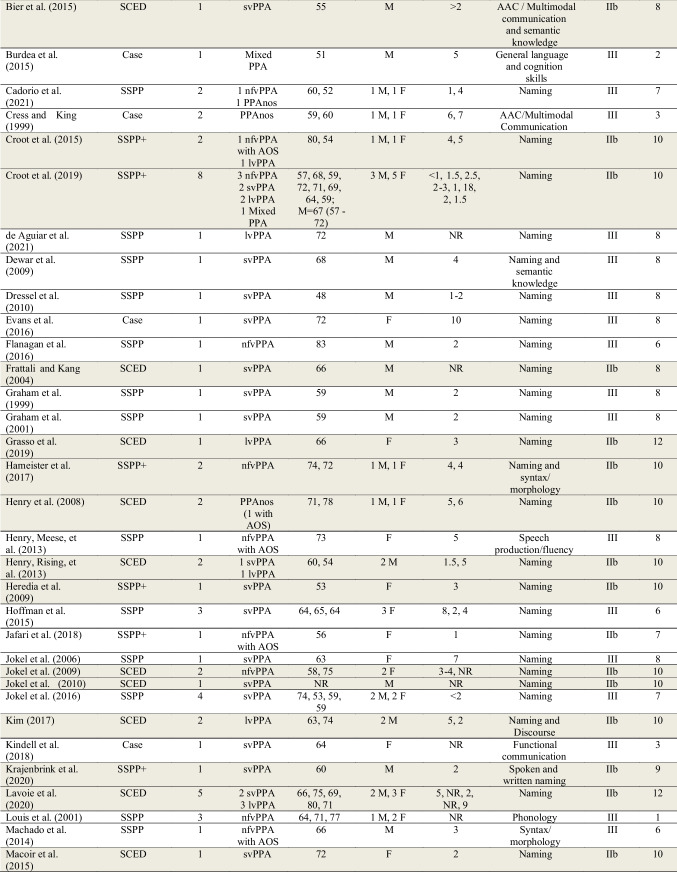

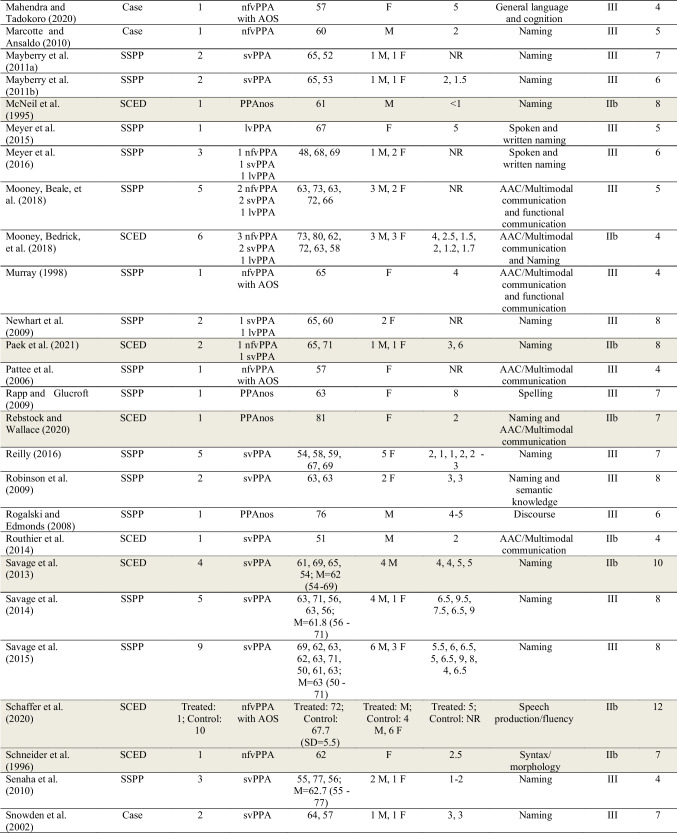

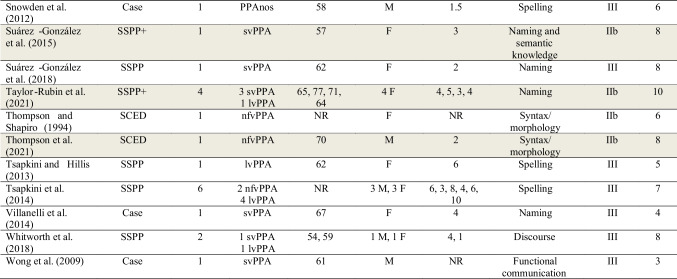
Summary characteristics of studies included in review

See Supplementary Materials 5 for the list of full references. Participants with the diagnostic label “semantic dementia” are categorized here as svPPA (semantic variant PPA). Participants with the diagnostic label “progressive nonfluent aphasia (PNFA)” are categorized here as nfvPPA (nonfluent variant PPA). Shaded studies are those included in the subset of 45 higher-quality studies. For further details, see Table 2 and “Results from the Higher-Quality Studies” section below

*PPA* Primary Progressive Aphasia, *lvPPA* logopenic variant PPA, *PPAnos* PPA not otherwise specified, *AOS* (apraxia of speech) in the PPA subtypes column indicates co-occurring AOS noted for at least one participant in the study, *SSPP* Single-subject Pre-post design, *SSPP*+ Single-subject Pre-post design meeting the criteria for level IIb (see “Methods”), *SCED* Single-case Experimental Design, *SD* Standard Deviation, *NR* Not reported, *M* Male, *F* Female, *LRT* Lexical Retrieval Treatment, *tDCS* transcranial direct current stimulation, *Naming* spoken naming of nouns, verbs, or adjectives unless otherwise noted, may include lexical retrieval (e.g., word finding) more broadly, *AAC* Augmentative and Alternative Communication, referring to a communication aid, book, or device that augments or provides an alternative to spoken communication, *Multimodal communication* utilizing various modes of communication (e.g., writing, drawing, gesturing) in addition to spoken communication, *Syntax/morphology* the sentence-level and word-level "grammar" of language

#### Participant Characteristics

The total number of study participants receiving behavioral treatment for speech or language across the 103 studies was 626. Of these, 207 had nfvPPA, 199 had svPPA, and 176 had lvPPA. Forty-two participants were considered to have PPA not otherwise specified, and two participants were considered to have mixed PPA. Twenty-nine participants in 12 studies were diagnosed with nfvPPA and noted to have AOS, but no studies used the diagnostic label “PPAOS.”

Regarding level of evidence for PPA diagnosis, 44 studies (42.7%) were rated as level 1 (the highest level of evidence; see Supplementary Materials 3). Twenty-nine studies (28.2%) were rated as level 2. Eighteen studies (17.5%) were rated as level 3. Seven studies (6.8%) were rated as level 4 and two (1.9%) as level 5. Three studies (2.9%) were given more than one rating (level 1 and 2, level 1 and 3, level 3 and 5) due to variability in the level of diagnostic information provided across participants to support stated diagnoses.

Levels of evidence for AOS diagnosis were generally lower. Of the 12 studies that included participants reported to have AOS, only one study (8.3%) was rated as level 1 (the highest level of evidence; see Supplementary Materials 4); one study (8.3%) was rated as level 3; eight studies (66.7%) were rated as level 4; and two studies (16.7%) were rated as level 5. See Supplementary Materials 6 for a list of all diagnostic evidence ratings for PPA and PPAOS.

Seventy-six studies analyzed data at the single-subject level. Forty-four (57.9%) of those reported data from a single participant; 67 (88.2%) from 5 or fewer participants. Twenty-seven studies analyzed data at the group level (mean number of participants = 17.5; SD = 11.1; range = 4–40).

Of the studies reporting the sex of PPA participants, 292 (46.6%) were male, and 297 were female (47.4%). The sex of nine participants was not reported, and the sex of 28 participants was uncertain because demographic data were reported across participants with PPA and other dementias. The mean age of participants in group design studies was 67.1 years (SD = 7.3; mean age for participants of one study not available) and in single-subject design studies was 64.4 years (SD = 7.6). Time post-onset of symptoms was inconsistently reported. Across the 27 group design studies, a mean and standard deviation for time post-onset was calculable for 15 studies (55.6%): mean time post-onset = 4.6 years; SD = 3.0. Of the 76 single-subject design studies, 64 (84.2%) reported time post-onset, although data were omitted for some participants. Some studies reported approximate ranges (e.g., “2–3 years”) and some a single value. The total number of participants for whom a single value for time post-onset was provided was 117: mean time post-onset = 4.0 years; SD = 2.6.

There was considerable heterogeneity in the reporting of additional participant sociodemographic, clinical history, speech, language, cognitive, and behavioral characteristics across studies. Further analysis of these is beyond the scope of the current review, but see the full table (Henry et al., 2023) for details (10.17605/OSF.IO/Z5U8D). A brief summary is provided in Supplementary Materials 10.

#### Treatment Targets

The greatest number of studies reported treatments targeting spoken naming/lexical retrieval (*k* = 67) or written naming/spelling (*k* = 16). Fewer studies targeted augmentative and alternative communication (AAC)/multimodal communication (*k* = 8), functional communication (i.e., communication strategy training or training for specific daily communication tasks; *k* = 7), semantic knowledge (*k* = 6), speech production/fluency (*k* = 6), spoken discourse production (*k* = 5), syntax/morphology (*k* = 5), general language and cognition (*k* = 3), word comprehension (*k* = 2), or phonology (*k* = 1).

### Study Designs and Levels of Evidence for Treatment Efficacy

**Fig. 2:**
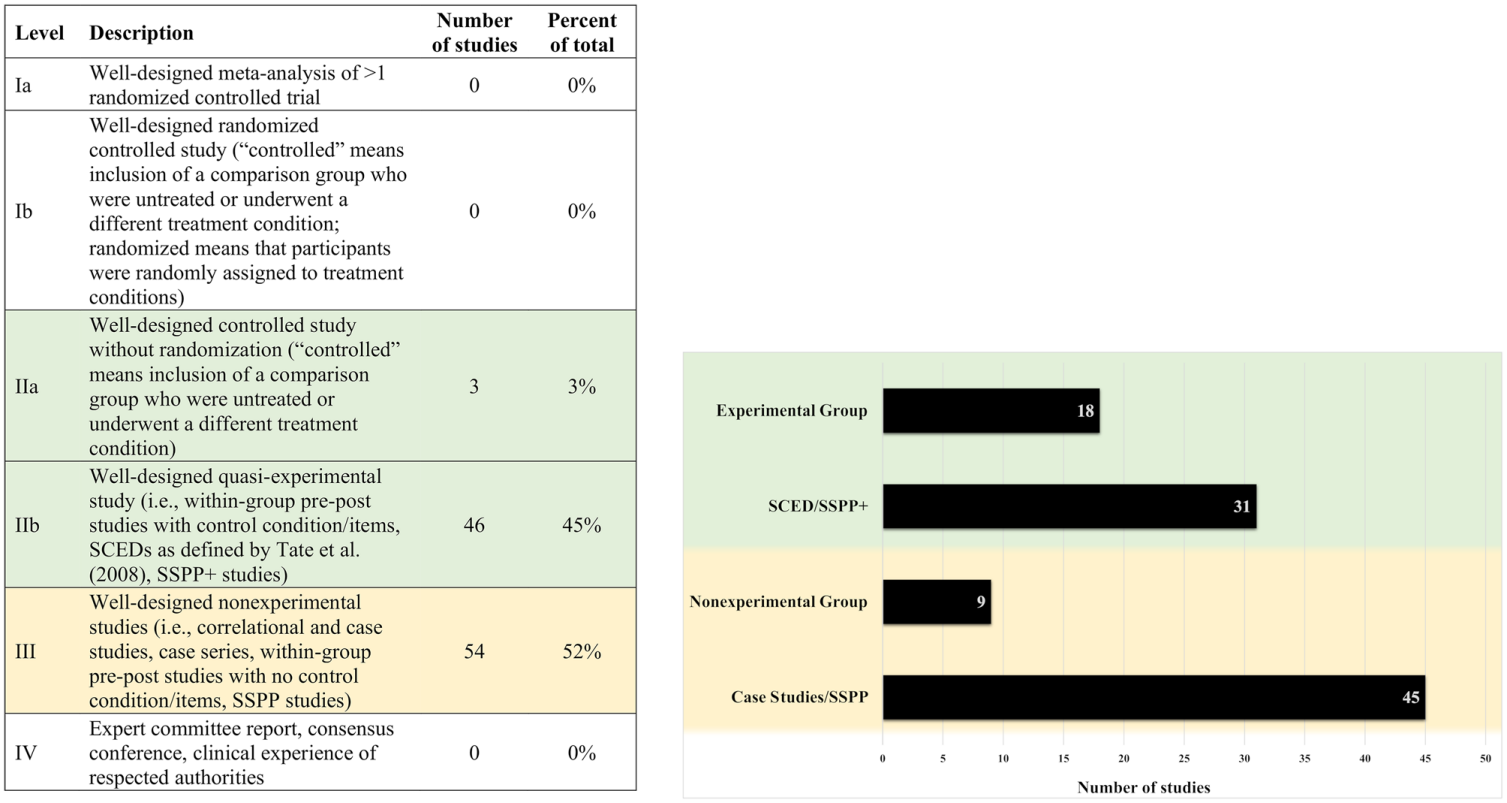
Number of studies with each design type and representing each of the levels of evidence adapted from the American Speech and Hearing Association (ASHA, 2004) and the Scottish Intercollegiate Guideline Network (Harbour & Miller, 2001). *SSPP*: single-subject pre-post design, *SSPP*+: single-subject pre-post design meeting criteria for Level IIb (see “Methods”), *SCED*: single-case experimental design

No studies met criteria for level I evidence. Three studies met criteria for level IIa, 47 for level IIb, and 53 for level III. Of the 50 experimental/quasi-experimental studies (levels IIa and IIb), 18 were group design studies and 32 were single-subject design studies. Of the 53 non-experimental studies, 9 were group design studies and 44 were single-subject design studies. See Fig. 2.

### Study Reporting Quality and Risk of Bias within Individual Studies

**Fig. 3:**
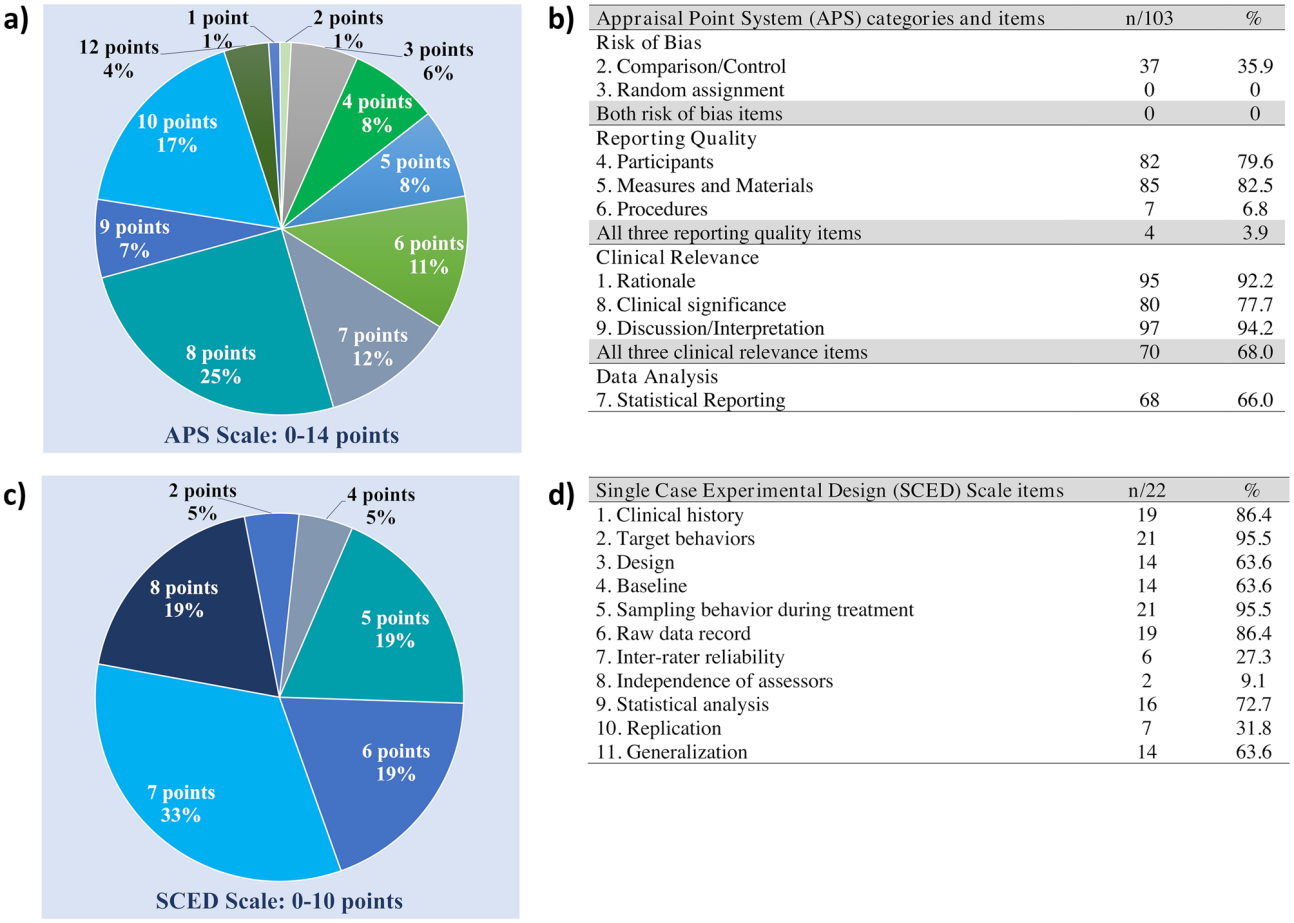
**a** Percentage of 103 studies achieving 1 to 12 points out of a maximum of 14 points on the appraisal point system (APS) items. **b** Number and percentage of studies rated positively for each APS item. **c** Percentage of 22 single-case experimental design (SCED) studies achieving 2 to 8 points out of a maximum of 10 points. **d** Number and percentage of SCED studies rated positively for each SCED scale item

Prior to the resolution of rater discrepancies via consensus, the proportion of itemized APS ratings with inter-rater agreement was 0.80; for SCED ratings was 0.85; and for PEDro-P ratings was 0.94. Itemized consensus APS scores for all 103 studies, SCED Scale scores for the 22 SCED studies (Tate et al., 2008), and PEDro-P scores for the three non-randomized controlled trials, as well as notes on scale items that had the lowest inter-rater agreement, are included in Supplementary Materials 7, Supplementary Materials 8, and Supplementary Materials 9, respectively. The percentage of studies receiving each APS total score is shown in Fig. 3a, and the number and percentage of studies receiving credit for each item is summarized in Fig. 3b. Sixty-five percent of studies received APS scores of 7 or greater. Less than half of studies (35.9%) were considered to have adequate comparison or control conditions on this scale. Statistical reporting was sufficient to receive credit in 66.0% of studies. Most studies reported adequately on participant characteristics (79.6%), and baseline/outcome measures and treatment materials (82.5%). Few (6.8%) reported adherence to the treatment protocol or treatment fidelity.

The percentage of studies receiving each SCED Scale total score is shown in Fig. 3c, and the number and percentage of studies receiving credit for each item is summarized in Fig. 3d. Among the 22 studies eligible for rating with the SCED Scale, the reporting and design elements that were typically present included sufficient details about participant clinical history (86.4%), description of target behaviors (95.5%), sufficient sampling of behavior (95.5%), and provision of the raw data record (86.4%). Elements that were present in fewer studies included inter-rater reliability (27.3%), independent evaluation of treatment outcomes (9.1%), and replication across participants, therapists, or settings (31.8%).

Three studies were rated using the PEDro-P scale. Two received scores of 5 and one received a score of 3 out of 10. None received points for randomization, concealed allocation, blinding of subjects, blinding of therapists, or blinding of assessors. All three specified eligibility criteria, obtained results from at least 85% of the participants initially enrolled, allocated participants to treatment or control conditions as specified, and statistically compared at least one outcome measure between groups. Two studies matched groups on important prognostic indicators, and two provided both a point measure and a measure of variability for at least one outcome.

Additional data on study reporting quality, internal validity, and external validity are provided in Supplementary Materials 11. Sixty-one percent of the studies described procedures that were replicable as reported. Twenty-one percent reported reliability measures for one or more outcome variables, and 34% replicated a treatment effect within the same study.

### Results from the Higher-Quality Studies

#### Study Designs

Forty-five studies used controlled study designs providing level IIa or level IIb evidence for treatment efficacy and achieved a score of 7 or greater on the APS (see shaded rows in Table 1), representing the higher-quality studies in the review. Treatment characteristics and outcomes of these studies are summarized in Table 2. Twenty-nine studies were classified as SCED or SSPP + , with 17 (58.6%) reporting data from a single participant; 28 (96.5%) studies had 5 or fewer participants. Sixteen studies were group experimental designs with 4 to 39 participants (mean = 15.5; SD = 7.7).

**Table 2:** Summary of outcome measures, treatment characteristics, and outcomes for the higher quality studies

	**Outcome measures**			**Treatment characteristics**				**Outcomes**			
**Reference**	**Primary**	**Generalization**	**Social validity**	**Treatment techniques**	**Mode of delivery**	**Frequency, duration, and no. of sessions with clinician**	**Frequency and duration of self-guided practice**	**Pri.**^**a**^	**Gen.**^**b**^	**Maint.**^**c**^	**Soc. Val.**^**d**^
**IIa group experimental designs**											
Dial et al. (2019)	LRT: Change score pre- to post-treatment for trained items. VISTA: % intelligible & correct words in each trained script topic	LRT: untrained words & BNT. VISTA: untrained script topics; NAT	NR	LRT: Cueing (semantic, orthographic, phonemic), repeat target, written copy target, semantic processing; VISTA – Script training, speech entrainment	One group in-person, one group tele-rehabilitation	LRT-1: 1 × /week, LRT-2: 2 × /week, VISTA: NR; Duration of sessions: NR (No. of sessions NR)	LRT: Modified Copy and Recall Treatment (CART) 1 × /day; VISTA: Speech entrainment 1 × /day for minimum 30 min	Y	Y	Y (12 mo)	NR
Farrajota et al. (2012)	Mean change in S&V naming test scores	token test, object naming, word repetition, comprehension of oral commands, object identification	NR	LRT: Attempted naming, semantic tasks, read, repeat and copy target, generate feelings, opinions, conversation	In-person	1 × /week for 60 min (Mean of 37.1 sessions)	Self-guided practice completed, but frequency is NR	Y	NR	NR	NR
Jokel et al. (2017)	Grades given by RAs for use of communication strategies	ASHA-QCL	ASHA -QCL, Qualitative feedback	LRT and functional communication training: Group education and practice for orthographic, phonological and semantic self-cueing; use of keywords, multimodal communication; Conversation practice with partner	In-person	1 × /week for 120 min (10 sessions)	NR	Y	NR	NR	Y
**IIb group experimental designs**											
Beales et al. (2021)	No. correctly named treated items; % CIUs & CIUs/min in connected speech samples	No. correctly named untreated items; ACE-III	NR	LRT: Self-cueing with autobiographical, semantic, orthographic and phonological cues; Application of cues in connected speech and conversation with a family member	In-person	3 × /week for 2 weeks, then 2 × /week for 4 weeks for 60–90 min (14 sessions)	Naming practice 2 × /week for 30 min; Conversation practice 1 × /day for 30 min	Y	Y	Y (6 wk)	NR
Cotelli et al. (2014)^e^	% correct naming of trained items	% correct naming of untrained items; Naming subtest of the AAT	SAQOL, CAS, Speech Questionnaire; Participant & family report	LRT: Repeat target, articulatory suppression task, attempted naming, read target word; administered in conjunction with tDCS or sham	In-person; computer-led	5 × /week for 25 min (10 sessions)	NR	Y	Y	Y (12 wk)	Y/N^f^
Cotelli et al. (2016)^g^	% correct naming of trained objects	% correct naming of untrained objects	SAQOL, CAS, Speech Questionnaire	LRT: Repeat target, articulatory suppression task, attempted naming, read target word; administered in conjunction with tDCS	In-person; computer-led	5 × /week for 25 min (10 sessions)	NR	Y	Y	Y (3 mo)	N
Flurie et al. (2020)	Accuracy of naming of trained items	Accuracy of naming of untrained items. Analysis of category-specific effects for objects vs. people; BNT, MoCA, PPT-W, PPT-P, Trails A, B, Digit span forward/back	NR	LRT: Attempted naming, semantic & phonological cueing, read and repeat target, semantic tasks	Caregiver-administered in the home; In-person or phone sessions led by clinician	1 × every 3 weeks; Duration of sessions: NR (No. of sessions NR)	Caregiver-led home practice 3 × /week; Duration of sessions: NR	Y	N	NR^g^	NR
Henry et al. (2018)	% correct, intelligible scripted words for trained script topics	% correct, intelligible scripted words for untrained script topics, proportion grammatical errors/100 words, overall intelligibility regardless of script content	In-house communication & communication confidence survey	Script training, speech entrainment, conversation practice	5 participants in-person, 5 participants tele-rehabilitation	2 × /week for 45–60 min (8–12 sessions, dependent on reaching 90% accuracy criterion for each trained script)	Speech entrainment and reading of difficult words/phrases 1 × /day for 30 min	Y	Y	Y (12 mo)	Y
Henry et al. (2019)	Accuracy of naming of trained items	Accuracy of naming of untrained items; BNT, WAB	In-house communication & communication confidence survey	LRT: Semantic, orthographic and phonological self-cueing in the presence of the picture, read, written copy, semantic plausibility judgement; recall to semantic cue	12 participants in-person, 6 participants tele-rehabilitation	LRT 1: 1 × /week for 60 min (Mean of 4.7 sessions) LRT 2: 2 × /week for 60 min (Mean of 11.4 sessions)	Modified Copy and Recall Treatment (CART) 1 × /day typically for ≤ 15 min	Y	Y	Y (12 mo)	Y
Hung et al. (2017)^h^	% accuracy for naming of trained items at the group & individual level	% accuracy for naming of untrained items at the group & individual level	NR	LRT: Repeat target and semantic features; sentence generation task; administered in conjunction with tDCS	In-person	1 × /day for 30 min (10 sessions)	NR	Y	N	N (6 mo)	NR
Jokel and Anderson (2012)	Naming accuracy on trained items overall & from each treatment condition	Recognition/comprehension of treated items; PNT, sentence production, structured connected speech, PPT, KDT, semantic fluency, PPVT	Participant report	LRT: Semantic/orthographic/phonological cueing, attempted naming or repeat target	In-person	2–3 days/week; twice each day for 30 min (96 sessions)	NR	Y	Y	Y (3 mo)	Y
Meyer et al. (2017)	% correct spoken naming for trained prophylaxis & remediation items	% correct spoken naming for unexposed exemplar pictures of trained items & % correct spoken naming for untrained, unexposed items; regional brain volumes	NR	LRT: read target in the presence of the picture, written copy of target; repeat target in the presence of the picture	For 18 Pts: Month 1: In-person; Months 2–6: In-person with clinician guidance 1 × /month and self-guided with caregiver assistance for the remainder; For 3 Pts: Tele-rehabilitation	For 18 Pts: 2 × /week over a month; 1 × /month over five months (Approximately 13 sessions) For 3 Tele-Rehab Pts: 2 × /week for first month then 3 × /week for next 5 months; Session Duration: NR (Approximately 68 sessions)	For 18 Pts: 3 × /week over five months following the month of sessions with a clinician; Session Duration: NR; For 3 Tele-Rehab Pts: NR	Y	Y	Y (1 mo)	NR
Meyer et al. (2019)	Mean change in naming accuracy for trained items & exemplars from phonologic & orthographic conditions	Mean change in naming accuracy for untrained items & exemplars from phonologic, orthographic conditions; naming accuracy in visual scene description	NR	LRT: read target in the presence of the picture, written copy of target; repeat target in the presence of the picture	For 23 Ps: Month 1: In-person; Months 2–6: In-person with clinician guidance 1 × /month and caregiver guidance for the remainder; For 3 Ps: Tele-rehabilitation	2 × /week for 45 min first month then 1 × /month for 45 min for five months (13 sessions)	After the first month of treatment, 3 × /week for 10–15 min for next 5 months (Typically 55 home practice sessions)	Y	Y	Y (8 mo)	NR
Meyer et al. (2016)	Accuracy of spoken naming of prophylactic or remediation items trained in either a phonological or orthographic treatment condition	Accuracy of spoken naming for untrained items; Accuracy of written naming for trained & untrained items; Naming of other exemplars; Naming accuracy in visual scene description	NR	LRT: read target in the presence of the picture, written copy of target, recognition task to ensure focus on stimuli; repeat target in the presence of the picture,	12 Participants In-person; 2 Participants Tele-rehabilitation	2 × /week for 45 min for first month; 1 × /month for 45 min for five months (13 sessions)	10–15 min of treatment tasks 3 × /week over five months (Approximately 60 sessions)	Y	Y	NR	NR
Roncero et al. (2019)^i^	No. trained items correctly named (S&V drawings) at final tDCS session for each of 3 tDCS conditions (parieto-temporal montage, DLPFC montage & sham)	No. untrained items correctly named (S&V drawings) at final tDCS session for each of 3 tDCS conditions; digit span forward/back, Letter Fluency, Animal fluency, MoCA, MMSE)	Observation of pt. mood	LRT: attempted naming, repeat in the presence of the picture, phonological or semantic cueing; administered in conjunction with tDCS or sham	In-person	For each round of stimulation, 2 × /week for up to 120 min for first week and 4 × /week for up to 120 min for two weeks, one month break in between rounds (30 sessions)	NR	Y	Y/N^i^	Y (2 mo)	Y
Zhao et al. (2021)^j^	Letter accuracy on written naming probes for trained items	Letter accuracy on written naming probes for untrained items	NR	LRT: attempted spoken & written naming, semantic feature generation, repeat in the presence of the picture, orthography to phonology task, written copy of target; administered in conjunction with tDCS or sham	In-person	5 × /week; Session duration: NR (Mean of 12 sessions)	NR	Y	Y/N^j^	Y (2 mo)	NR
**Single-subject experimental designs**											
Beales et al. (2016)*	Naming accuracy – treated items	Naming accuracy – untreated items; Curtin University Discourse Protocol	In-house communication & communication confidence survey	LRT: Self-cueing with autobiographical, semantic, orthographic & phonological cues	In-person	2 × /week for 90 min (8 sessions)	Minimum of 2 × /week for 30 min	Y	Y	Y (4 wk)	Y
Beeson et al. (2011)*	No. words in generative naming for trained categories	No. words in generative naming for untrained categories; Standardized test scores; Neuroimaging results	Self-assessment of change measures; Participant & family report	LRT: Read target aloud in the presence of the picture, semantic categorization/association tasks, generative naming in categories,	In-person	6 × /week for 2 h (12 sessions)	1 × /day for 60 min	Y	Y	Y (6 mo)	Y
Bier et al. (2015)	% learned smartphone functions	Naming accuracy & semantic feature generation for trained & untrained words	NR	Assistive device training: Errorless procedure rehearsal, practice search strategies with device	In-person	NR (5 sessions)	NR	Y	Y	Y (6 mo)	NR
Bier et al. (2009)	% correct on spoken picture naming & generation of semantic attributes for trained items	% correct on spoken picture naming & generation of semantic attributes for untrained items; letter fluency	NR	LRT: Repeat target in the presence of the picture and generation of semantic attributes, spaced retrieval training	In-person	2 × /week; Duration of sessions: NR (6 sessions)	Instructed not to practice at home	Y	N	Y (5 wk)	NR
Bier et al. (2011)	% correct ability to follow steps to prepare & cook a meal	Frequency of using SemAssist & cooking in the home; Generation of semantic attributes of ingredients in trained & untrained recipes; knowledge of food in the context of food preparation; DO-80 Naming Test	Participant & family report of frequency of cooking meals	Assistive device training: use of SemAssist computer program & internet search engines; Semantic feature generation	In-person	Approximately every other week; Duration of sessions: NR (8 sessions)	Encouraged to practice the target recipe using SemAssist which logged the number of times it was accessed (logs show she used it between 2–27 times per month)	Y	N	Y (6 mo)	Y
Croot et al. (2015)	Accuracy of naming trained words	Accuracy of naming of untrained words. Use of naming skills within connected speech	Participant & family report	LRT: Repeat & read target in the presence of the picture	Self-guided computer practice (Clinician present for first session)	1 (Remainder were self-guided)	5 × /week for 10–15 min over 2 weeks	Y	Y	N (1 mo)	Y
Croot et al. (2019)	Proportion correct naming of trained items	Proportion correct naming of untrained items	NR	LRT: Repeat & read target in the presence of the picture	Self-guided computer practice (Clinician present for first session)	1 (Remainder were self-guided)	5 × /week for 10 min over 2 weeks	Y	N	Y (54 wk)	NR
Frattali and Kang (2004)	% correct naming of trained items	% correct naming of untrained items; Object & Action Naming Battery; WAB; Generative naming, BNT, S&V Naming; PALPA subtests; DRS- 2, CADL-2	CADL- 2; ASHA-QCL	LRT: Semantic tasks, attempted naming	In-person	1 × /week for 2 h (12 sessions)	NR	Y	N	N (3 mo)	N
Grasso et al. (2019)	% correct responses on probes for each set of trained words	% correct responses on probes for each set of untrained words. BNT	Participant & family report	LRT: Semantic tasks, phonological cueing, read or read & repeat target in the presence of the picture, generative naming	In-person sessions led by clinician or caregiver	2 × /week for 60 min (8 sessions with the clinician; 4 sessions with the caregiver)	Modified Copy and Recall Treatment (CART) 1 × /day for approximately 30 min; Optional practice with flashcards (frequency NR)	Y	Y	Y (12 mo)	Y
Hameister et al. (2017)	% correct retrieval of nouns & verbs in picture description, % correct reduced sentence structures, % correct complete sentence structures (in picture description & spontaneous speech samples)	CAT, GNT, TROG-2, video retell	NR	Constraint-Induced Aphasia Therapy: production of verb phrases, card game and LRT:self-cueing with phonological cueing hierarchy	In-person (group therapy)	4–5 × /week for 60 min (9 sessions	Computer-based practice 1 × /day for 30 min	Y	Y	Y (2 mo)	NR
Henry et al. (2008)	No. items in generative naming for trained categories	No. items in generative naming for untrained categories, WAB, BNT, PPT	NR	LRT: Semantic tasks; Read target in the presence of the picture; Generative naming	In-person	1 × /day for 90 min (12 sessions)	1 × /day	Y	Y	Y (4 wk)	NR
Henry et al. (2013)	No. correctly named trained items	No. correctly named untrained items. WAB, BNT, PPT, MMSE	In-house communication & communication confidence survey; Participant & family report	LRT: Semantic, orthographic & phonological self-cueing in the presence of the picture, read, written copy, semantic plausibility judgement; recall to semantic cue	svPPA: In-person; lvPPA: tele-rehabilitation	svPPA: phase1: 2 × /week for 60 min (8 sessions) phase 2: 5 × /week for 120 min (12 sessions); lvPPA: 1–2 × /week for 60 min (6 sessions)	svPPA: Phase 1: Naming practice 5 × /week for 30 min; Phase 2: 1 × /day for 60 min. lvPPA: Modified Copy and Recall Treatment (CART) for 3 h/week	Y	Y	Y (4 mo)	Y
Heredia et al. (2009)	No. correctly named trained items	No. correctly named untrained items; No. correctly named items that were visually similar exemplars of trained items	Spouse report	LRT: Attempted naming, read target in the presence of the picture	Self-guided computer practice with help from spouse as needed	N/A (Self-guided practice)	1 × /day over the course of 1 month; Duration of sessions: NR	Y	Y	Y (6 mo)	Y
Jafari et al. (2018)	No. correctly named trained items	No. correctly named untrained items	NR	LRT: Attempted naming, semantic cueing, phonological cueing; Story production	In-person	2 × /week for 60 min (16 sessions)	NR	Y	Y	Y (2 wk)	NR
Jokel et al. (2009)	% correct spoken naming of trained items	PNT, sentence production task (% correct)	NR	LRT: Orthographic cueing, read or read & repeat in the presence of the picture	Computer-based with clinician supervision	Case 1: 3 × /week for 60 min (12 sessions); Case 2: 2 × /week for 60 min (36 sessions)	NR	Y	Y	Y (4 wk)	NR
Jokel et al. (2010)	% correct spoken naming of trained items	% correct spoken naming of untrained items. PNT, oral sentence production test, PPT (words & pictures), KDT, verbal fluency	Quality of Communication Life Scale	LRT: Semantic task, read or read & repeat target in the presence of the picture	Computer-based with clinician supervision	3 × /week for 60 min (30 sessions)	NR	Y	Y	Y (3 mo)	Y
Kim (2017)	% correct spoken naming for trained words; % CIUs	% correct spoken naming for untrained items; Mean No. of well-formed sentences	NR	LRT: Semantic tasks, generate autobiographical information, generate orthographic & phonological cues, read target aloud, written copy of target, picture description	In-person	Both phases: 2 × /week for 50 min (Phase 1: 19 sessions; Phase 2: 23 sessions)	Copy and Recall Treatment: 1 × /day; Duration: NR	Y	Y	Y (5 mo)	NR
Krajenbrink et al. (2020)	No. correct on spoken & written naming for trained items	No. correct responses on a picture-word verification task; No. words retrieved in response to structured interview questions	NR	LRT: Read & repeat & written copy target in the presence of the picture; semantic tasks	Self-guided computer practice	N/A (Self-guided)	Phase 1: 5 × /week for no more than 10 min over two weeks; Phases 2 and 3: 5 × /week over two weeks; Session Duration: NR	Y	Y	N (2 wk)	NR
Lavoie et al. (2020)	No. correct on spoken naming for trained items	No. correct on spoken naming for untrained items; %age of times pt. displayed anomia for trained & untrained items during conversation task	NR	LRT: Semantic tasks, attempted naming, repeat target in the presence of the picture	Self-guided tablet application practice	N/A (Self-guided)	4 × /week for 45–120 min over four weeks	Y	Y	Y (2 mo)	NR
Macoir et al. (2015)	% correct spoken naming of treated verbs	% correct spoken naming of untreated verbs	NR	LRT: Semantic cueing, phonological cueing	In-person	2 × /week for 5 weeks then 1 × /week for 2 weeks; Length of sessions: NR (12 sessions)	NR	Y	N	Y (4 wk)	NR
McNeil et al. (1995)^k^	Accuracy providing antonyms or synonyms to established adjective lists	No. of items generated for related items within (e.g. adjectives) & between word classes; Performance on the Porch Index of Communicative Ability; Revised Token Test, CPM, Rapid Automatized Naming Test, DDK, Discourse measures (No. of words/min, CIUs/min, % CIUs, Average pause duration, % total words by class)	NR	LRT: Generate synonyms or antonyms, gestural cueing, semantic & phonological cueing, written word recognition, repeat target; administered in conjunction with a pharmacological agent and a withdrawal condition	In-person	1 × /week for 120 min (31 sessions)	NR	Y	Y	Y (1 mo)	NR
Paek et al. (2021)	No. correctly named trained action & object items	No. correctly named untrained action & object items; No. correctly named unexposed action & object items; % CIUs in a narrative & a procedural discourse; TAAWF, TONI-3, CTONI-2, MMSE-2	CETI	LRT: semantic tasks, phonological tasks, memory game, charades, card game, attempted naming, semantic & phonological cueing, repeat target in the presence of the picture	In-person	At least 2 × /week for 60 min (16 sessions)	NR	Y	Y	Y (3 mo)	Y
Rebstock and Wallace (2020)	No. correct spoken naming for treated words; Communicative Flexibility score (No. of modality switches relative to opportunities to switch as a %age) on the Referential Communication Task	No. of untreated words in Naming; Listener accuracy in judging initial production (any modality) during Referential Communication Task; WAB-R, CADL-2, PALPA 48 Written Word to Picture Matching, PALPA 51 Word Semantic Association, PALPA 52 Spoken Word to Written Word Matching; CLQT; WCST	Referential communication task	LRT: semantic feature analysis, repeat target in the presence of the picture; Multimodal communication training: imitate naming, gesturing and drawing the target concept	In-person	4 × /week for 120 min followed by a week-long break, then 4 × /week for 120 min (8 sessions)	30 min of “semantically based home practice” after each treatment session	Y	Y	NR	Y
Savage et al. (2013)	Accuracy of spoken naming for trained words	Accuracy of spoken naming for untrained words	NR	LRT: repeat and read target in the presence of the picture, sentence generation task	Self-guided computer practice	N/A (Self-guided)	Instructed to practice 1 × /day for 30–60 min over six weeks	Y	N	Y (2 mo)	NR
Schaffer et al. (2020)	% correct intelligible, scripted words for trained topics	% correct intelligible, scripted words for untrained topics; overall speech intelligibility (% intelligible words during script probes regardless of targeted script content); grammatical complexity (ratio complex grammatical relations: total No. of grammatical relations); speech rate in WPM; MLU in morphemes & words	In-house communication & communication confidence survey; CETI	Script training, speech entrainment, conversation practice	Tele-rehabilitation	2 × /week for 45–60 min (12 sessions)	30 min/day of unison speech production practice with video; Maintenance period: Encouraged to continue with home practice	Y	N	Y (12 mo)	Y
Schneider et al. (1996)	% grammatically correct verbal & gestural responses to line drawings for trained verb tenses	% correct gestural responses for untrained verb tenses; Discourse measures on Cinderella retell: Total words, MLU, % grammatical sentences, % simple sentences, % complex sentences, % conjoined sentences, average no. embedded clauses/sentence, noun:verb ratio, open:closed class ratio, verb morphology index, & % correct verb morphology index	NR	Verb tense training: repeat spoken target, copy gestural target	In-person	12 sessions (Frequency and duration of sessions: NR)	NR	Y	Y	Y (3 mo)	NR
Suárez-González et al. (2015)	Naming accuracy for trained words	Naming accuracy for untrained words; Naming accuracy for novel exemplars of trained items; Providing a description of each trained item; Naming trained items following experimenter description	NR	LRT: attempted naming, read target; Conceptual enrichment therapy: semantic association task, generate autobiographic information	For both phases: 1 in-person session and then self-guided computer practice with occasional telephone check-ins from clinician	1 × /phase (2 sessions)	For both phases: Instructed to complete therapy steps 1 × /day for at least 30 min over three months	Y	Y	NR	NR
Taylor-Rubin et al. (2021)	No. treated nouns & verbs in confrontation naming; No. verb phrases containing treated nouns & verbs in picture description task	No. untreated nouns & verbs in confrontation naming; No. correct verb phrases containing untreated nouns & verbs in picture description task	NR	LRT: Repeat & read target in the presence of the picture	Self-guided computer practice	N/A (Self-guided)	5 × /week over two weeks; Session duration: NR	Y	Y	NR	NR
Thompson et al. (2021)	% correct passive sentences & object cleft sentences on structured probes; % correct on sentence matching task using targeted sentence forms	NNB, PPT, NAVS, NAVI, Cinderella Narrative Analysis; Pre/post eye tracking data (e.g., proportion of fixations to target picture for comprehension; timing of gazes to agent & theme during production); Pre/post fMRI data (e.g., uptake in activation in ROIs for syntactic comprehension)	NR	Treatment of underlying forms: physical manipulation of argument structure of verbs, thematic/syntactic mapping from canonical (e.g., active voice) to noncanonical (e.g., passive voice) forms	In-person	2 × /week for 90 min for two phases of 12 weeks (48 sessions)	NR	Y	Y	Y (1 yr)	NR

^a^Primary outcome measure, *Y* indicates positive outcome on a primary outcome measure for at least one individual, ^b^Generalization measure, *Y* indicates positive outcome on a generalization measure for at least one individual; ^c^Maintenance, *Y* indicates maintenance of treatment outcomes observed at any timepoint after the end of treatment designated as “maintenance” or “follow-up”, interval at which maintenance is reported is also given: *wk* week/s, *mo* month/s, ^d^Social validity, *Y* indicates social validation of treatment gains reported for at least one individual, *N* No, *N/A* not applicable, *No.* number, *NR* not reported, *CART* copy and recall treatment, *VISTA* video implemented script training for aphasia, *LRT* lexical retrieval treatment, *BNT* Boston Naming Test (see studies included in review for details of tests as cited in the original studies), *WAB* Western Aphasia Battery, *PNT* Philadelphia Naming Test, *PPT* Pyramids and Palm Trees, *PPVT* Peabody Picture Vocabulary Test, *S&V* Snodgrass & Vanderwart, *tDCS* transcranial direct current stimulation, *MoCA* Montreal Cognitive Assessment, *MMSE* Mini-Mental Status Examination, *DO-80* Test de dénomination orale d’images, *CADL- 2* Communication Activities of Daily Living 2nd Edition, *ASHA-QCL* American Speech and Hearing Association Quality of Communication Life Scale, *PALPA* Psycholinguistic Assessments of Language Processing in Aphasia, *DRS* Dementia Rating Scale, *CAT* Comprehensive Aphasia Test, *GNT* Graded Naming Test, *TROG* Test of Reception of Grammar, *KDT* Kissing and Dancing Test, *CPM* Colored Progressive Matrices, *DDK* diadochokinesis, *CIU* content information unit, *TAAWF* Test of Adolescent and Adult Word Finding, *TONI* Test of Nonverbal Intelligence, *CTONI* Comprehensive Test of Nonverbal Intelligence, *CLQT* Cognitive Linguistic Quick Test, *WCST* Wisconsin Card Sorting Test, *WPM* words per minute, *MLU* mean length of utterance, *CETI* Communicative Effectiveness Index, *NNB* Northwestern Naming Battery, *NAVS* Northwestern Assessment of Verbs and Sentences, *NAVI* Northwestern Assessment of Verb Inflection, *fMRI* functional magnetic resolution imaging, *ROIs* regions of interest, ^e^Administered behavioral therapy in conjunction with tDCS or a sham. Improvements in the primary outcome, generalization to untrained items, and maintenance were found in both groups with some increased effects for the tDCS group. Evidence of a socially valid effect of treatment was found only in the tDCS group; ^f^Administered behavioral treatment in conjunction with tDCS, there was no sham comparison group. ^g^Treatment is described as a “maintenance-based” treatment, but the main treatment continued throughout the duration of the study, so maintenance as defined here was not measured. ^h^Administered behavioral treatment in conjunction with tDCS; there was no sham comparison group. ^i^Administered behavioral treatment in conjunction with two tDCS conditions or a sham condition. Improvements in the primary outcome and maintenance of these effects compared to baseline were found for all three conditions. Generalization to untrained items was found only for one of the tDCS conditions. Qualitative evidence of socially valid treatment effects was broadly described and it is unclear whether there were differences between groups on this outcome. ^j^Administered behavioral treatment in conjunction with tDCS or sham. Improvements in the primary outcome and maintenance of these improvements were found in both groups with some increased effects for the tDCS group. Generalization to untrained items was found only for the tDCS group. ^k^Administered behavioral treatment alone and in conjunction with a pharmacological agent. Improvement in the primary outcome, generalization, and maintenance were found in both conditions

#### Participants

The total number of treated participants across the higher-quality studies was 304: 110 had nfvPPA, 93 had svPPA, 96 had lvPPA, 4 had PPAnos, and 1 had Mixed PPA. Sixteen participants had AOS, but none was diagnosed with PPAOS. One hundred and forty (46.1%) were male and 155 (51.0%) were female; the sex of nine participants was not reported. The mean age of participants in the group design studies was 67.1 years (SD = 7.5) and in single-subject design studies was 66.2 years (SD = 7.7). Of the 16 group design studies, a mean and standard deviation for time post-onset was calculable for 8 studies (50.0%); this was 4.5 years (SD = 3.8). Twenty-seven of the single-subject design studies (93.1%) reported time post-onset. A single time post-onset value was reported for 46 participants; average time post-onset for these participants was 3.7 years (SD = 2.7).

#### Treatment targets and techniques

In the 45 studies, spoken naming/lexical retrieval was the most frequent target of treatment (37 studies, 82.2%). Four studies (8.9%) targeted semantic knowledge, and three studies (6.7%) each targeted syntax/morphology, speech production/fluency, and written naming/spelling. Discourse, functional communication, and AAC/multimodal communication were each targeted by two studies (4.4%). One study (2.2%) targeted word comprehension.

Lexical retrieval treatment techniques included errorless learning (i.e., eliciting a target response from the participant under conditions that minimize the opportunity to produce an incorrect response), cueing or self-cueing to aid retrieval (e.g., phonological, orthographic, semantic or autobiographical self-cues), generative naming (i.e., generating a list of words from a target category), generating synonyms and antonyms, and conversational practice with a communication partner. Other treatment approaches included script training (i.e., developing and practicing personally relevant scripts for use in conversation), constraint-induced aphasia therapy (i.e., an intensive therapy approach in which spoken communication is reinforced and use of other modalities is minimized), training use of an assistive device (e.g., using a smart phone app for accessing semantic information about foods in a recipe), written naming treatment, training multimodal communication (i.e., augmenting verbal communication with other modalities such as writing or gesturing), and the explicit training of morphosyntactic structures (e.g., physically manipulating visual representations of syntactic structure in a sentence and rehearsing spoken production of the target structure).

#### Treatment Delivery

Twenty-seven of the higher quality studies (60%) provided intervention in person (Table 2); some (8 studies, 17.8%) provided a combination of limited in-person sessions with self**-**guided or caregiver-administered therapy. Seven studies (15.6%) used self-guided practice as the primary mode of therapy. Five (11.1%) provided telerehabilitation (therapy via videoconferencing), two (4.4%) compared results following in-person therapy with results following caregiver-administered therapy or telerehabilitation. Six (13.3%) provided both in-person therapy and telerehabilitation but did not investigate differences in outcomes across modes.

Regarding the number of sessions completed with a clinician, 10 studies (22.2%) reported fewer than 10 sessions; 17 (37.8%) reported 10–25 sessions; and 7 (15.5%) reported more than 25 sessions. Four studies (8.9%) included participants or groups who received differing amounts of treatment. The remaining seven studies (15.6%) relied on self-guided or caregiver-guided practice only. The frequency of treatment sessions across studies varied widely: often sessions were held twice weekly, however, in some studies as frequently as six times weekly or twice per day on 2–3 days per week. Twenty-eight studies (62.2%) incorporated independent practice to be completed outside of clinician-led sessions: 21 provided independent activities supporting skills addressed in clinician-led sessions, and seven used self-guided practice as the primary intervention, as noted above.

#### Outcome Measures

The most frequent primary outcome measure was spoken or written confrontation naming (33/45 studies, 73.3%; see Table 2). Several other primary outcome measures were used by a smaller number of studies, including grammatical correctness/complexity, discourse informativeness/efficiency, generative naming, script accuracy, naming in connected speech, and use of AAC or alternate modes of communication. Generation of semantic attributes, accuracy of completing activities of daily living (ADLs), ratings of functional communication, and generation of synonyms/antonyms were primary outcome measures in one study each. Generalization data included measures of the target behaviors for untrained items, for different exemplars of treated items, or in connected speech, as well as performance on standardized tests (e.g., general tests of language or cognition or tests evaluating target behaviors such as lexical retrieval). Nineteen studies addressed social validity, most often relying on the qualitative report of the participant or the participant’s family (9 studies); others reported functional communication measures (6 studies), in-house surveys (6 studies), quality of life measures (3 studies), or clinician observations (1 study).

#### Treatment Outcomes

**Fig. 4:**
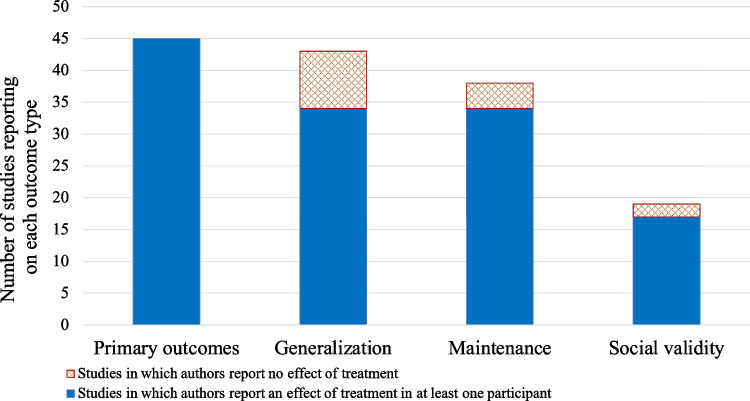
Summary of outcomes of behavioral treatment from the 45 higher-quality* studies identified by the systematic review. Bar height reflects the number of studies reporting on the primary outcome measure, generalization, maintenance, or social validity of treatment. Blue color indicates the number of studies reporting a positive outcome for at least one participant, and orange color with diamond pattern indicates the number of studies reporting no effect of treatment (*Experimental and quasi-experimental studies with adequate reporting and acceptable risk of bias following the criteria of Oren et al., 2014)

A summary of treatment outcomes across the 45 higher-quality studies is provided in Fig. 4, according to whether they reported a positive result on a primary outcome measure, generalization measure, measure of maintenance of treatment gains, or social validity measure for at least one participant in each study. Authors of the reviewed studies reported a positive result for a primary outcome measure for at least one participant in all 45 studies (100%). Forty-three studies investigated generalization, 34 of which (79.1%) reported a positive result on a generalization measure for at least one participant. Thirty-eight studies reported outcomes for one or more time points designated as “maintenance” or “follow-up” time points, 34 of which (89.5%) reported maintenance of treatment gains for at least one participant. Of the 19 studies reporting social validity outcomes, 17 (89.5%) reported a positive result on a social validity measure for at least one participant.

Five of the higher-quality studies examined behavioral treatment in conjunction with tDCS, and one study administered treatment in conjunction with a pharmacological agent. A post hoc summary of outcomes excluding groups or conditions with a neuromodulatory or pharmacological adjunct to treatment was compiled to establish outcomes of behavioral treatment alone. Because two of the studies with a tDCS adjunct did not include a sham condition, only 43 studies were included in the summary. All 43 studies (100%) reported a positive result for a primary outcome measure. Of 41 studies reporting on generalization, 31 (75.6%) showed a positive result. Of 36 studies reporting on maintenance, 33 (91.7%) showed a positive result. Of 18 reporting on social validity, 16 (88.9%) showed a positive result.

Given the high rates of positive results across all four outcome types for at least one participant per study, a post hoc summary of outcomes for every participant across the higher-quality single-subject design studies was compiled to establish whether these positive results were consistent across all participants (results for every participant were not available for the experimental group studies). There were 29 single-subject design studies that reported results for 56 participants. In those studies, a positive result on a primary outcome measure was reported for 53 individuals (94.6%). Generalization was investigated for all 53 of these participants, and 33 (62.3%) showed a positive result on a generalization measure. Maintenance results were reported for 46 of these 53 participants and 39 (84.8%) showed maintenance of gains. Social validity was reported for 18 participants, and 17 (94.4%) showed socially valid results of treatment.

## Discussion

### Summary of Evidence

The first question addressed by this systematic review was “What evidence exists to support behavioral treatment for speech or language in PPA and PPAOS, and what is the quality and credibility of this evidence?” A total of 103 studies published between 1994 and 31 May 2021 were identified that investigated behavioral treatment for speech or language in PPA. This represents an almost threefold increase in the number of studies since the first systematic review of cognitive rehabilitation outcomes in PPA in 2013 (Carthery-Goulart et al., 2013). Twelve studies reported on treatment for AOS in persons diagnosed with PPA, but no treatment studies for persons given a diagnosis of PPAOS were identified by the review. The highest-quality and most credible evidence is currently provided by 45 level IIa and level IIb (experimental and quasi-experimental) studies with adequate reporting and acceptable risk of bias following the criteria of Oren et al. (2014): 16 experimental group studies and 29 single-subject designs.

The second question was whether behavioral treatment results in improvement on a primary outcome measure, generalization to untrained targets/behaviors/environments, maintenance of gains, or socially-validated outcomes. Of the 45 higher-quality studies, all reported improvement on a primary outcome measure for at least one participant. The majority of these studies also reported at least one participant with generalization and maintenance of treatment gains. Just under half of the higher-quality studies addressed social validity, with most of these reporting improvement on a social validity measure for at least one participant. Similar positive results were found following a post hoc analysis across all participants in the higher-quality single-subject design studies. This systematic review therefore supports the provision of behavioral treatment for speech and language for persons with PPA.

Below we discuss the interventions for PPA identified by the review and the results for treatment generalization, maintenance, and social validity. We also discuss study design issues identified by the current review, limitations of the review, and clinical implications.

### Treatments Supported by This Review

Lexical retrieval treatment was particularly well supported by the available evidence, with 82.2% of the higher-quality studies reporting improvement on target behaviors following treatment for naming or lexical retrieval. The remaining higher-quality studies offer support for interventions targeting skills other than lexical retrieval, including the use of AAC and other forms of multimodal communication, functional communication strategies, grammatical processing, the fluent production of speech, and discourse/conversation skills. The high proportion of lexical retrieval treatment studies likely reflects the pervasiveness of anomia in PPA (Mesulam et al., 2003; Sebastian et al., 2018), and the robust evidence base for anomia treatment in the stroke-induced aphasia literature (Nickels, 2002). It also represents an emphasis on restitutive or impairment-directed treatment, targeting body structures and functions within the World Health Organization International Classification of Functioning, Disability, and Health (WHO-ICF, 2001).

Information regarding staging of treatment, or the relative utility of specific types of intervention for patients of different severity levels, was not addressed in the current review. However, studies suggest that impairment-directed interventions, including lexical retrieval treatment, may be most effective earlier in the disease course (for example, better lexical retrieval treatment outcomes are seen in individuals with less severe aphasia (e.g. Savage et al., 2015). In the early stages of PPA, prophylactic treatment also may be appropriate to slow the decline of intact skills rather than to regain skills that have already been diminished (Meyer et al., 2015, 2016, 2019). Interventions that are participation-directed (focused on activities or social participation) and environment-directed (focused on communication partners and contexts) may be especially relevant in middle-to-late stages of the disease, when technology or communication partners are increasingly required to support the individual’s declining functional communication (see, for example, Mahendra & Tadokoro, 2020).

Narrative reviews have long advocated for proactive anticipation of likely future care needs (Rogers et al., 2000) and for regular reassessment as needs change with decline (Sapolsky et al., 2011), but only two studies in the current review described a treatment approach that adapted with PPA disease trajectory (Mahendra & Tadokoro, 2020; Murray, 1998). Mahendra and Tadokoro described how palliative care principles guided the selection of behavioral interventions (including impairment-directed, participation-directed, and environment-directed treatment components) over a 3-year period to optimize both communication and quality of life for one individual. Additionally, Murray reported a 2.5-year longitudinal study that moved from language stimulation through the use of a drawing program to communication partner training, to adapt to the changing communication abilities and needs of the person with PPA.

### Treatment for PPAOS

No studies reported on treatment for individuals with a diagnosis of PPAOS. Possible explanations include the apparently lower prevalence of PPAOS compared to PPA, the relatively recent recognition of PPAOS as a distinct diagnostic entity, and the fact that current PPA diagnostic criteria (Gorno-Tempini et al., 2011) allow for a diagnosis of nfvPPA when AOS is present and aphasia is not (i.e., PPAOS is captured within nfvPPA). Nonetheless, AOS was reported in 29 participants across 12 studies (16 participants in five higher-quality studies). In four studies (Henry et al., 2013, 2018; Schaffer et al., 2020; Themistocleous et al., 2021), two from the subset of higher-quality studies (Henry et al., 2018; Schaffer et al., 2020), speech production was a treatment target, AOS was a targeted disorder, and positive treatment results were reported. This review demonstrates a need, and some promise, for treatment addressing progressive AOS. Future treatment studies for nfvPPA will be informative with regard to PPAOS treatment if they explicitly report the presence/absence of AOS and, where speech production is a treatment target (for example, script training studies), if motor speech planning/programming abilities are directly evaluated and treated.

### Generalization, Maintenance, and Social Validity Outcomes

Most of the higher-quality studies included in this review investigated generalization or maintenance of treatment gains in addition to changes in trained skills and targets, and the majority of these reported positive results. The selection of generalization measures and time point(s) for evaluating maintenance varied widely across studies (see below).

The following generalization measures were reported by study authors: performance on untrained words, language structures, or topics that were closely matched to trained targets; the use of target behaviors in discourse or the home environment; performance on speech, language, or cognitive tasks not directly related to the treatment task; and naming of novel picture exemplars of trained items. In our main analysis, we considered generalization to have been demonstrated in a study if the authors reported improvement on at least one generalization measure for at least one individual. Our results, therefore, indicate where there is promise of generalization of treatment outcomes; however, theoretically motivated investigation of the types of generalization observed, relative to specific participant characteristics and types of treatment, is warranted. For example, treatments delivered in the context of functional tasks, or in a person’s everyday environments, may facilitate better generalization to settings outside the clinic.

This review also observed maintenance of treatment gains, consistent with an earlier systematic review that found maintenance of treatment gains across PPA subtypes at intervals from one to six months (Cadório et al., 2017). For studies in the current review, the intervals at which maintenance was measured varied considerably across studies, ranging from 2 weeks to 33 months. Furthermore, studies varied regarding whether participants had ongoing access to practice materials or received “treatment boosters” during the maintenance period. As a consequence, it is difficult to draw firm conclusions about the length of time individuals with PPA can be expected to maintain treatment effects with or without subsequent practice, and treatment maintenance mechanisms await systematic investigation.

Fewer than half of the higher-quality studies (42.2%) reported measures of social validity, including functional communication and quality of life scales as well as qualitative observations by researchers, family members, and the participants themselves. None of the scales or measures have been validated for use in PPA; therefore, development and validation of such measures for this population is a priority for future research. Given that holistic approaches to therapy emphasize the importance of addressing the goals of persons with PPA and their close others, future studies should consider identifying those goals consistently and evaluating their attainment as a method of characterizing social validity.

### Study Design Considerations

Studies in the present review used varying criteria to establish diagnosis, in part reflecting the changing diagnostic framework for PPA over the last 30 years. For example, prior to the publication of the PPA consensus criteria (Gorno-Tempini et al., 2011), individuals with lvPPA may have been diagnosed with another PPA subtype, such as PNFA (Henry & Gorno-Tempini, 2010). Additionally, groups have differed with regard to whether semantic dementia may be considered equivalent to svPPA when object recognition impairments are present (Mesulam et al., 2014). Additional subtypes beyond those in the consensus criteria have also been described (e.g., Marshall et al., 2018). The impact of varied diagnostic criteria on the aggregation of data across studies could be minimized with consistent reporting of information to establish cognitive-linguistic profiles and support diagnostic reporting. However, the ratings for level of evidence for PPA diagnosis in this review showed that fewer than half of studies (42.7%) provided speech-language and neuropsychological assessment data adequate to support the PPA diagnosis and subtype, with some (6.8%) only providing a diagnostic label. Future studies should carefully document individual participant factors including age; level of education; time post-onset; and speech, language, cognitive, and behavioral profiles so that multi-site collaborative projects or meta-analyses can begin to identify predictors of treatment response in larger samples.

There were no RCTs that provided level I evidence for behavioral treatment efficacy under the modified ASHA evidence classification scheme. While there are a handful of RCTs in the PPA treatment literature (e.g., Harris et al., 2019; Tsapkini et al., 2018), all used neuromodulation and randomized participants either to active stimulation versus sham conditions, or to different orders of sham/stimulation in crossover designs. These studies provide level I evidence regarding the utility of neuromodulation as an adjunct to behavioral treatment (see Cotelli et al., 2020), but do not provide the same level of evidence regarding efficacy of behavioral treatment per se. Several factors account for the absence of RCTs in this review. First, the low prevalence of PPA and PPAOS makes it difficult to recruit sufficiently large samples for an adequately powered prospective group study running two or more groups in parallel. Multi-center (see published protocol for a RCT currently underway; Roberts et al., 2022) and international collaborations will likely be required to achieve the required sample sizes. Alternatively, different types of intervention designs, such as Bayesian adaptive designs, hold promise, with the ability to evaluate different treatment characteristics within limited sample sizes. Second, RCT design requires a solid understanding of the variables to be manipulated or controlled, given the costs of RCTs and multicenter trials. The high-quality single-subject design studies (SCED and SSPP +) identified in the current review provide a cost-effective way to establish the set of variables to be manipulated and controlled in RCTs (Levine & Downey-Lamb, 2002) and a rich resource for the design of future RCTs. SCED and SSPP + studies are especially important in low-prevalence and progressive syndromes such as PPA and PPAOS, where information on participant and treatment factors associated with positive treatment results can accrete over time on a study-by-study basis. Third, selecting an appropriate control group for a RCT is challenging. No-treatment control groups raise ethical concerns for speech or language treatment for persons with PPA/PPAOS, since asking individuals with a progressive condition to abstain from treatment even for a short period of time may deny them the opportunity to benefit from therapies while they are still able. Only two group studies in this review used a no-treatment control group: one used a historical control group (Farrajota et al., 2012) and the other a waitlist control group (Jokel et al., 2017). These types of controls avoid the ethical concerns associated with prospective no-treatment control designs, but demographic matching of treatment and control participants can be difficult to accomplish, and outcome data must be collected under equivalent conditions to avoid confounds that may render group comparisons invalid. Instead, trials comparing such treatment factors as dosage (more versus less intensive), nature of training stimuli (e.g., personalized versus generic), or training parameters (e.g., errorless versus errorful training) may be most appropriate for this clinical population.

In total, fewer than half of all studies (45/103) met our criteria for inclusion in the subset of higher-quality studies for which we summarized treatment characteristics and outcomes (level I or II on the modified ASHA levels of evidence classification scheme and an APS score of 7 or higher). The APS, SCED, and PEDro-P ratings indicated areas of strength and weakness in the design and reporting of all 103 studies. General strengths were the quality of reporting on participants, target behaviors, measures and materials, raw data records, and clinical relevance. A minimum of two of three statistical reporting criteria (appropriate statistical analysis, inclusion of descriptive statistics, and inclusion of effect sizes; APS item #7) was met by only two-thirds of studies, and inclusion of adherence or protocol fidelity measures, use of study designs with adequate experimental control, and randomization of participants to groups were achieved by fewer studies, representing areas of weakness. It should be noted, however, that many SCED and SSPP + design studies randomize the allocation of items, rather than participants, to conditions, establishing a level of experimental control not recognized by the APS. Among the studies with designs that permitted rating with the SCED scale, the failure to address inter-rater reliability, independence of assessors, and replication of results (in at least one other participant or environment) were areas of weakness. PEDro-P ratings indicated that the controlled group studies demonstrated weaknesses in randomization; concealed allocation; and blinding of subjects, therapists, and assessors. Together, these weaknesses highlight aspects of study design and statistical reporting and analysis that should be rigorously addressed in the future.

### Limitations of the Review

The limitations of this review relate to the instruments we used to characterize reporting quality, risk of bias, and levels of evidence for treatment efficacy; the approaches we took to characterizing diagnosis and treatment outcomes; the scope of interventions for PPA that we reviewed; and the restriction of our review to studies published in English.

#### Critical Appraisal Tools

We note limitations of the selected instruments used to characterize methodological and reporting quality and level of evidence. Following the methodology of previous systematic reviews conducted by the ANCDS (Ballard et al., 2015; Wambaugh et al., 2006), we used the SCED (Tate et al., 2008) and PEDro-P (Murray et al., 2013) scales for rating methodological quality for single-subject and experimental group studies, respectively. These tools are limited with regard to study design types that can be appraised. SCED is intended for use only with multiple baseline, ABA, alternating treatment, and changing criterion designs (Tate et al., 2008) and PEDro-P for use only with RCT and nonrandomized controlled trials (Murray et al., 2013). We anticipated significant heterogeneity of study designs, including quasi-experimental studies not eligible for SCED or PEDro-P ratings. Therefore, we also implemented the APS (Oren et al., 2014), because it provides a common framework for evaluating study quality across heterogeneous study designs.

To assess the level of evidence for treatment efficacy, we modified the ASHA levels of evidence for treatment efficacy scheme (ASHA, 2004), which was originally adapted from the SIGN framework (Harbour & Miller, 2001). Although this rating scheme is consistent with that used in previous systematic reviews, we found that it lacked sufficient specificity and granularity to delineate level II (quasi-experimental) versus level III (non-experimental) single-subject studies, which represented approximately half of the studies identified for our review. As such, we further operationalized the criteria for single-subject designs to be designated as quasi-experimental versus non-experimental (see “Methods”). Both the APS and the modified ASHA levels of evidence framework have limitations. Their validity and reliability have not been systematically evaluated, and the APS instrument is limited in its appraisal of risk of bias, particularly when compared to more recently-developed instruments, such as the *JBI Critical Appraisal Checklist for Quasi-Experimental Studies* (non-randomized controlled studies; “JBI Manual for Evidence Synthesis,” 2020) or the *Risk of Bias in N-of-1 Trials Scale* (RoBiNT; Tate et al., 2013).

#### Characterization of Treatment Outcomes

We reported the proportion of the 45 higher-quality studies in which the authors indicated a positive result for at least one participant on at least one primary outcome measure. This analysis allowed us to discern which interventions show promise, as a guide to both clinical practice and intervention research, but there is a risk that it oversimplified complex or mixed results. We therefore also completed a post hoc participant-by-participant summary of outcomes for the higher-quality subset of single-subject designs (where participant-by-participant data were available for scrutiny). The results of the post-hoc summary largely corroborated the results of the main summary but did reveal a slightly lower proportion of participants showing positive results from treatment (94.6% on a primary outcome measure, 62.3% on a generalization measure, 84.8% on a maintenance measure, 89.5% on a social validity measure) as compared to the proportion of studies documented in the main summary (100% on a primary outcome measure, 79.1% on a generalization measure, 89.5% on a maintenance measure, 94.4% on a social validity measure).

We did not conduct a meta-analysis. We aimed to provide a comprehensive overview of behavioral intervention for speech and language in PPA, and we identified a range of study designs addressing a variety of research questions. Characterizing these designs and questions represents the first step toward determining which emerging techniques (e.g., Shadish et al., 2016) would be suitable for aggregating data across studies, and which questions can potentially be answered using meta-analysis. This, in turn, would indicate which data should be extracted from which studies in future meta-analyses. Cotelli et al. (2020) and Nissim et al. (2020) have already reported meta-analyses of spoken and written naming treatment outcomes following behavioral treatment with and without tDCS; and Roheger et al. (2021) have published a protocol for a review of word retrieval and quality of life outcomes in PPA as part of the Cochrane Collaboration. With regard to the wide range of other interventions not targeting lexical retrieval, the Volkmer et al. (2020a) systematic review commented on the impossibility of aggregating data from studies with incommensurate outcome measures, and the same difficulties would apply to this review. Future meta-analyses may address questions about particular treatment approaches, types of generalization, maintenance of treatment gains, or predictors of treatment response.

In the absence of standardized effect sizes, we were also unable to quantify reporting/publication bias across studies. This will be an important future step in systematic appraisal of the treatment literature in PPA/PPAOS. We advocate for careful reporting of participants who are excluded or withdraw from experimental or quasi-experimental treatment studies and for publication of null results to minimize the risk of reporting and publication bias in the literature.

#### Scope of Interventions Reviewed

This review focused on interventions directed at speech or language in individuals with PPA/PPAOS, inclusive of interventions targeting communication more broadly (e.g., multimodal communication or other augmentative and alternative communication strategies). It excluded interventions directed at care partners and family members, with the exception of studies providing communication-related interventions for care partners together with persons with PPA (e.g., Jokel et al., 2017; Mooney et al., 2018). Care partners of persons with PPA/PPAOS are challenged with learning about a complex and relatively rare disorder as well as meeting new and evolving procedural and logistical demands required for caregiving (Reinhard et al., 2008; Schumacher et al., 2000). Additionally, they may face pre-existing or emerging disability associated with their own health conditions, or “third party disability” (Organization, 2001) (i.e., the negative health consequences related to the burdens and stressors imposed by caregiving). Failure to address these issues may affect the care partner’s ability to provide care and support to the person with PPA/PPAOS (Reinhard et al., 2008). At present, a limited number of studies have begun to address the need for caregiver-focused interventions (Schaffer & Henry, 2021); these should be considered in future reviews of the treatment literature in PPA/PPAOS.

The review similarly excluded interventions directed specifically toward psychological well-being for persons with PPA, although speech or language interventions with outcome measures characterizing changes in communication-related quality of life, confidence, and mood were captured. Anxiety, depression, frustration, and grief are common in persons with PPA (Davies & Howe, 2019; Ruggero et al., 2019) and interventions targeting psychological well-being are warranted; at present, however, only a small number of such interventions have been reported (Morhardt et al., 2019; Schaffer & Henry, 2021; Taylor-Rubin et al., 2019).

#### Restriction to Studies Published in English

We restricted the review to studies published in English because we did not have access to multi-language searching or to translation resources. Beyond reducing the total number of eligible studies, this almost certainly reduced the representation of individuals speaking languages other than English.

### Implications for Behavioral Treatment for PPA and PPAOS in Clinical Practice

Our review identified 45 experimental and quasi-experimental (level IIa and IIb) studies with adequate study quality and risk of bias documenting positive treatment outcomes in PPA. To understand the clinical implications of these results, it is useful to compare our results to those of the previous comprehensive systematic review of behavioral speech-language treatment in PPA (i.e., Carthery-Goulart et al., 2013). Carthery-Goulart and colleagues (2013) used a different evidence characterization system (Cicerone et al., 2000) to classify studies by class of evidence and synthesize overall evidence for a given type of intervention into one of three practice recommendations: practice standards, practice guidelines, or practice options. According to the most recent review by Cicerone and colleagues (2019), practice standards are supported by at least one prospective RCT or treatment study with quasi-randomized allocation to treatment condition (class I studies), providing “substantive” evidence of effectiveness; practice guidelines are supported by class I studies with methodological limitations or “prospective, nonrandomized cohort studies, retrospective, nonrandomized case–control studies, or multiple baseline design studies that permitted a direct comparison of treatment conditions” (class II studies) providing evidence of “probable” effectiveness; and practice options are supported by class II studies or “clinical series without concurrent controls or single-subject designs with adequate quantification and analysis” (class III studies), providing evidence of “possible” effectiveness (Cicerone et al., 2019, p. 1517). Carthery-Goulart and colleagues (2013) recommended lexical retrieval treatment as a practice option for svPPA and noted there were a handful of other treatments supported by one or two class III studies.

The current systematic review identified no RCTs; thus, none of the intervention approaches can be classified as a practice standard using the Cicerone et al. (2019) recommendation system. Our findings included three prospective nonrandomized cohort studies and several multiple baseline design studies that documented positive outcomes across PPA variants following lexical retrieval treatment, all of which qualify as class II evidence according to Cicerone and colleagues (2000, 2019). The evidence for lexical retrieval treatment in this review therefore best supports an updated designation as a practice guideline for all subtypes of PPA. Furthermore, our findings included one prospective randomized cohort study, one multiple baseline design study, and one well-designed group study without concurrent controls documenting positive outcomes following script training in individuals with nfvPPA. Taken together, this evidence is likely commensurate with a recommendation as a practice guideline for nfvPPA, although it will be important for more research groups to replicate the current positive results. All other interventions in the group of higher-quality studies in the current review (e.g., multimodal communication training, assistive device training; see “Results”) met criteria for recommendation as practice options.

Evidence-based clinical recommendations for treatment of individuals with PPAOS are sparse; however, a few higher-quality studies addressing progressive AOS in individuals with nfvPPA document positive outcomes (e.g., Henry et al., 2018, documents positive outcomes following script training). Until there is a larger evidence base, clinical decision-making and provision of behavioral treatment for PPAOS and progressive AOS in the context of nfvPPA must also rely on treatment efficacy evidence for nondegenerative AOS (Ballard et al., 2015), as well as clinical experience, expert opinion, and general principles guiding treatment for other degenerative motor speech disorders (e.g., principles of motor learning; Duffy et al., 2020).

## Conclusions

The number of peer-reviewed journal articles reporting behavioral treatment for speech or language in PPA has almost tripled in the 8 years between the first systematic review (Carthery-Goulart et al., 2013) and our most recent search. There is a large proportion of high-quality studies (45/103), and these indicate that a variety of treatment approaches may result in positive treatment results for trained targets as well as generalization and maintenance of outcomes, with a small number of studies also providing evidence of social validity. This review updates the clinical recommendations of Carthery-Goulart et al. (2013) with additional practice guidelines and options to inform clinical practice. These results strongly support physician referral of individuals with PPA to a speech-language pathologist-therapist and implementation of appropriate behavioral intervention.

This review also indicates where further research is warranted. Treatments identified as practice guidelines and practice options should be investigated using study designs with the potential to provide level I and level II evidence of efficacy. Mechanisms of improvement, generalization, and maintenance, as well as factors predicting individual outcomes, require systematic investigation, the results of which will guide the selection of participants and treatment approaches most suitable for future randomized controlled trials.

## Supplementary Information

Below is the link to the electronic supplementary material.

Supplementary file1 (PDF 31.5 KB)

Supplementary file2 (PDF 96.2 KB)

Supplementary file3 (PDF 76.2 KB)

Supplementary file4 (PDF 111 KB)

Supplementary file5 (PDF 168 KB)

Supplementary file6 (PDF 128 KB)

Supplementary file7 (PDF 194 KB)

Supplementary file8 (PDF 4.8 KB)

Supplementary file9 (PDF 75.9 KB)

Supplementary file10 (PDF 98.9 KB)

Supplementary file11 (PDF 20 KB)

## Data Availability

The dataset generated by the systematic review are available via the Open Science Framework at 10.17605/OSF.IO/Z5U8D.

## Abbreviations

AAC: Augmentative and alternative communication
AOS: Apraxia of speech
APS: Appraisal point system
lvPPA: Logopenic variant primary progressive aphasia
nfvPPA: Nonfluent variant primary progressive aphasia
PEDro-P: Physiotherapy Evidence Database – PsycBITE Rating Scale for Randomized and Non- Randomized Controlled Trials
RCT: Randomized controlled trial
PPA: Primary progressive aphasia
PPAOS: Primary progressive apraxia of speech
SCED: Single-case experimental design
SSPP: Single-subject pre-post design
SSPP+: Single-subject pre-post designs that included at least two measurements of the target behavior in the baseline phase, a statistical analysis of change across study phases accounting for both measurements in the baseline phase, and a control condition or control items
svPPA: Semantic variant primary progressive aphasia
tDCS: Transcranial direct current stimulation
