# Supplementary material for: Behavioral Treatment for Speech and Language in Primary Progressive Aphasia and Primary Progressive Apraxia of Speech: A Systematic Review
Source: Neuropsychol Rev. 2023 Oct 4;34(3):882–923. doi: 10.1007/s11065-023-09607-1 (PMC11473583; doi:10.1007/s11065-023-09607-1)
Supplement: Supplementary file 1 — Supplementary file1 (PDF 31.5 KB) [file 11065_2023_9607_MOESM1_ESM.pdf]

Wauters, L.D., Croot, K., Dial, H.R., Duffy, J.R., Grasso, S.M., Kim, E., Schaffer, K.M., Ballard, K.J., Clark, H.M., Kohley, L., Murray, L.L., Rogalski, E.J., Figeys, M., Milman, L., Henry, M.L., Behavioral treatment for speech and language in primary progressive aphasia and primary progressive apraxia of speech: A systematic review**.** *Neuropsychology Review.*

Corresponding author: Maya Henry, Department of Speech, Language, and Hearing Sciences, The University of Texas at Austin, 2504A Whitis Ave. (A1100), Austin, TX 78712-0114,
Email: maya.henry@austin.utexas.edu.

Supplementary Materials 1: *Estimated prevalence of primary progressive aphasia and primary progressive apraxia of speech reported in this review*

Prevalence of 3.66 per 100,000 estimated from Coyle-Gilchrist et al. (2016)**:** Crude prevalence calculated for this manuscript based on 62 individuals with nfvPPA, svPPA or other PPA in a sample of 200 cases identified from specialist clinics, clinical research networks, advertisement, self-referral and relevant charities in two English counties in 2013 and 2014.

Estimated prevalence of 3.23 per 100,000 reported by Magnin et al. (2016)**:** Prevalence for the total French population based on French National AD Database consultations for nfvPPA, lvPPA, svPPA and unclassifiable PPA between May 2012 and May 2013

Estimated prevalence of 3.54 per 100,000 estimated from Borroni et al.(2010)**:** Crude prevalence calculated for this manuscript based on 12 semantic dementia and 31 PNFA (progressive nonfluent aphasia) cases in a sample of 213 in the Italian county of Brescia from January 2001 to December 2008, estimating number of cases lost by the census date for each PPA subtype by multiplying the total enrolled for each subtype (13 semantic dementia and 33 PNFA) by the proportion remaining of the total enrolled sample at census data (.943).

Estimated prevalence of PPAOS close to 2 per 100,000 by Botha & Utianski (2020)**.** Based on the prevalence of PPAOS relative to other speech and language disorders in one major cohort and reported prevalence of other speech and language disorders. Note that a significant proportion of individuals classified as PPAOS in these studies [31,113] likely had overlapping clinical presentations with those characterized as nfvPPA in studies used to estimate PPA prevalence here.
