# Supplementary material for: Behavioral Treatment for Speech and Language in Primary Progressive Aphasia and Primary Progressive Apraxia of Speech: A Systematic Review
Source: Neuropsychol Rev. 2023 Oct 4;34(3):882–923. doi: 10.1007/s11065-023-09607-1 (PMC11473583; doi:10.1007/s11065-023-09607-1)
Supplement: Supplementary file 2 — Supplementary file2 (PDF 96.2 KB) [file 11065_2023_9607_MOESM2_ESM.pdf]

Wauters, L.D., Croot, K., Dial, H.R., Duffy, J.R., Grasso, S.M., Kim, E., Schaffer, K.M., Ballard, K.J., Clark, H.M., Kohley, L., Murray, L.L., Rogalski, E.J., Figeys, M., Milman, L., Henry, M.L., Behavioral treatment for speech and language in primary progressive aphasia and primary progressive apraxia of speech: A systematic review**.** *Neuropsychology Review*.

Corresponding author: Maya Henry, Department of Speech, Language, and Hearing Sciences, The University of Texas at Austin, 2504A Whitis Ave. (A1100), Austin, TX 78712-0114, E-mail: maya.henry@austin.utexas.edu.

Supplementary Materials 2: *Summary of systematic reviews of treatment for people with primary progressive aphasia.*

**Author, year**	**Scope of review (Publication years of reviewed studies)**	**No. studies reviewed (No. pwPPA reported)**	**Main conclusions of review**		
			**Immediate gains**	**Generalisation**	**Maintenance**
Carthery-Goulart et al. (2013)	Establish evidence-based recommendations for cognitive rehabilitation in PPA (1995-2013)	39* (69)	All studies showed gains. Impairment-directed lexical retrieval treatment recommended as a Practice Option for svPPA; insufficient evidence (low numbers of Class III studies) to allow other recommendations.	svPPA: not to untreated items or treated stimuli in different context, with exceptions.<br>nf/avPPA: most generalised gains to some degree to untreated items, tasks, functional communication<br>lvPPA: generalisation to untreated items, conversation	svPPA: maintenance for variable periods after treatment.<br>nfvPPA & lvPPA: no general conclusion
Cadorio et al. (2017)	Analyse effects of semantic therapy on generalisation and maintenance of treatment in PPA (2002-2015)	25 (51)	All studies showed gains	svPPA: a few reports, in tasks very similar to treatment task; also overgeneralisation of new learning<br>nfvPPA & lvPPA: more common than in svPPA. Transfer from L2 to L1 in one lvPPA	Maintenance above baseline levels at one-to-six months post-therapy in all variants
Cotelli et al. (2020)	Efficacy of language training (2001-2019) or language training with rTMS or	43 language training (239); 7 language training with tDCS (111)	Language training alone or with tDCS improves oral naming of trained items; language training with tDCS	Language training with tDCS improves oral and written naming of untrained items	Language training with tDCS improves written naming of untrained items at follow-up

	tDCS (2014-2018) on oral and written naming accuracy in PPA		improves written naming of trained items		(variable from 1-144 weeks across studies)
Volkmer et al. (2020)	Identify functional communication interventions for pwPPA & caregivers, their effectiveness, & key intervention components (1998-2018)	19 (91)	All studies showed gains; key components are building on strategies currently used by pwPPA and practice with a communication partner	n/a	n/a

Notes: PPA = primary progressive aphasia, pw = people with, sv = semantic variant, nfv = nonfluent variant, lv = logopenic variant, rTMS = repetitive transcranial magnetic stimulation, tDCS = transcranial direct current stimulation, L1 = a speaker's first or native language, L2 = a speaker's second (non-native) language, *Includes 36 studies of behavioral treatment for speech/language in PPA.
