# Supplementary material for: Behavioral Treatment for Speech and Language in Primary Progressive Aphasia and Primary Progressive Apraxia of Speech: A Systematic Review
Source: Neuropsychol Rev. 2023 Oct 4;34(3):882–923. doi: 10.1007/s11065-023-09607-1 (PMC11473583; doi:10.1007/s11065-023-09607-1)
Supplement: Supplementary file 3 — Supplementary file3 (PDF 76.2 KB) [file 11065_2023_9607_MOESM3_ESM.pdf]

Wauters, L.D., Croot, K., Dial, H.R., Duffy, J.R., Grasso, S.M., Kim, E., Schaffer, K.M., Ballard, K.J., Clark, H.M., Kohley, L., Murray, L.L., Rogalski, E.J., Figeys, M., Milman, L., Henry, M.L., Behavioral treatment for speech and language in primary progressive aphasia and primary progressive apraxia of speech: A systematic review**.** *Neuropsychology Review.*

Corresponding author: Maya Henry, Department of Speech, Language, and Hearing Sciences, The University of Texas at Austin, 2504A Whitis Ave. (A1100), Austin, TX 78712-0114, E-mail: maya.henry@austin.utexas.edu.

Supplementary Materials 3: *Levels of evidence for the diagnosis of PPA*

This rating indicates the degree to which the participant's or participants' speech-language and cognitive characteristics are consistent with consensus definitions of PPA (e.g., Mesulam, 2001) and PPA subtype (e.g., Gorno-Tempini et al., 2011).

a. **Level 1:** PPA diagnosis and subtype diagnosis are supported by adequate assessment information: speech, language, and cognitive data presented in support of PPA diagnosis. No exclusionary features documented (e.g., early, prominent visuospatial or behavioral changes). If non-speech/language impairments noted, they must **not** have been early and prominent features. To be considered a Level 1 study, there must be sufficient cognitive and speech-language data to document that speech-language deficits were/are greater than cognitive deficits. Additionally, adequate information must be given to establish subtype diagnosis relative to stated criteria (does **not** need to be Gorno-Tempini, et al., 2011). This can include participants who are "mixed" or do not neatly fit a subtype.
b. **Level 2:** Adequate information to confirm PPA diagnosis, but not for diagnosis by subtype. Incomplete diagnostic information for subtyping (e.g., statement that the participant met diagnostic criteria, but insufficient assessment data provided) or exclusionary features for stated subtype noted.
c. **Level 3:** Inadequate diagnostic information to confirm PPA diagnosis or subtype; no exclusionary behaviors/features.
d. **Level 4**: Stated diagnosis of PPA and/or subtype, but NO description of characteristics in support of the diagnosis.
e. **Level 5:** Descriptions that are contradictory to the stated criteria for diagnosis of PPA and PPA subtype. Or NO DIAGNOSTIC CRITERIA INDICATED.
