# Supplementary material for: Behavioral Treatment for Speech and Language in Primary Progressive Aphasia and Primary Progressive Apraxia of Speech: A Systematic Review
Source: Neuropsychol Rev. 2023 Oct 4;34(3):882–923. doi: 10.1007/s11065-023-09607-1 (PMC11473583; doi:10.1007/s11065-023-09607-1)
Supplement: Supplementary file 4 — Supplementary file4 (PDF 111 KB) [file 11065_2023_9607_MOESM4_ESM.pdf]

Wauters, L.D., Croot, K., Dial, H.R., Duffy, J.R., Grasso, S.M., Kim, E., Schaffer, K.M., Ballard, K.J., Clark, H.M., Kohley, L., Murray, L.L., Rogalski, E.J., Figeys, M., Milman, L., Henry, M.L., Behavioral treatment for speech and language in primary progressive aphasia and primary progressive apraxia of speech: A systematic review**.** *Neuropsychology Review.*

Corresponding author: Maya Henry, Department of Speech, Language, and Hearing Sciences, The University of Texas at Austin, 2504A Whitis Ave. (A1100), Austin, TX 78712-0114, E-mail: maya.henry@austin.utexas.edu.

Supplementary Materials 4: *Levels of evidence for the diagnosis of AOS*

This rating indicates the degree to which the participant's or participants' speech characteristics are consistent with the definition of AOS provided by McNeil, Robin, & Schmidt (1997) and the diagnostic descriptors as provided below (Wambaugh et al., 2006).

a. **Level 1:** All of the primary characteristics consistent with the definition of AOS. Any characteristics that are attributable to other disorders may be described but are not used to diagnose AOS. No exclusionary behaviors are described.
b. **Level 2:** All of the primary characteristics consistent with definition of AOS and some speech characteristics that may be attributable to aphasia or dysarthria. No exclusionary behaviors are described.
c. **Level 3:** Most of the primary characteristics consistent with definition of AOS and some speech characteristics that may be attributable to aphasia or dysarthria. No exclusionary behaviors are described.
d. **Level 4:** Incomplete/inadequate description of the discriminative characteristics. No exclusionary behaviors are described.
e. **Level 5:** Stated diagnosis of AOS, but no description of characteristics consistent with that diagnosis and descriptions that are contradictory to the criteria for diagnosis.

Diagnostic Descriptors of AOS (Wambaugh et al., 2006)

Clinical Characteristics – Primary

- slow speech rate: lengthened segments (vowels and/or consonants)
- slow speech rate: lengthened intersegment durations (sounds, syllables, words, phrases; possibly filled, as in the case of an intrusive schwa)
- sound distortions (including consonants and vowels)
- distorted sound substitutions
- errors are relatively consistent in type (e.g., substitution, omission, distortion) and location in repeated utterances
- prosodic abnormalities

Clinical Characteristics – Nondiscriminative

- articulatory groping – audible and/or visible and probably distorted relative to target
- perseverative errors (perseverations of movement patterns)
- increasing errors with increasing word length
- speech initiation difficulties
- awareness of errors (e.g., self-corrections)
- automatic speech better than propositional speech
- islands of error free speech

Clinical Characteristics that Cannot be Used to Diagnose AOS

The following characteristics have been used to diagnose AOS in the past. However, they are more likely attributable to other disorders. Their presence is not necessarily contradictory to a diagnosis of AOS, because they may occur in conjunction with AOS.

- anticipatory errors
- transposition errors
- limb or oral non-speech apraxia
- expressive-receptive speech/language gap

Exclusionary Characteristics

- fast rate
- normal rate
- normal prosody

Additional Considerations

Severe AOS can be diagnosed by mutism or extremely limited repertoire of speech sounds in conjunction with relatively preserved language skills and in the absence of abnormal nonspeech oral functions for vegetative purposes and volitional movement.
