# Supplementary material for: Behavioral Treatment for Speech and Language in Primary Progressive Aphasia and Primary Progressive Apraxia of Speech: A Systematic Review
Source: Neuropsychol Rev. 2023 Oct 4;34(3):882–923. doi: 10.1007/s11065-023-09607-1 (PMC11473583; doi:10.1007/s11065-023-09607-1)
Supplement: Supplementary file 5 — Supplementary file5 (PDF 168 KB) [file 11065_2023_9607_MOESM5_ESM.pdf]

Wauters, L.D., Croot, K., Dial, H.R., Duffy, J.R., Grasso, S.M., Kim, E., Schaffer, K.M., Ballard, K.J., Clark, H.M., Kohley, L., Murray, L.L., Rogalski, E.J., Figeys, M., Milman, L., Henry, M.L., Behavioral treatment for speech and language in primary progressive aphasia and primary progressive apraxia of speech: A systematic review**.** *Neuropsychology Review.*

Corresponding author: Maya Henry, Department of Speech, Language, and Hearing Sciences, The University of Texas at Austin, 2504A Whitis Ave. (A1100), Austin, TX 78712-0114, E-mail: maya.henry@austin.utexas.edu.

Supplementary Materials 5: *Reference list for all studies included in the systematic review*

Suárez-González, A., Savage, S. A., & Caine, D. (2018). Successful short-term re-learning and generalisation of concepts in semantic dementia. *Neuropsychological Rehabilitation*, *28*(7), 1095–1109. https://doi.org/10.1080/09602011.2016.1234399

de Aguiar, V., Zhao, Y., Ficek, B. N., Webster, K., Rofes, A., Wendt, H., Frangakis, C., Caffo, B., Hillis, A. E., Rapp, B., & Tsapkini, K. (2020). Cognitive and language performance predicts effects of spelling intervention and tDCS in Primary Progressive Aphasia. *Cortex, 124*, 66–84. https://doi.org/10.1016/j.cortex.2019.11.001

Evans, W. S., Quimby, M., Dickey, M. W., & Dickerson, B. C. (2016). Relearning and retaining personally-relevant words using computer-based flashcard software in primary progressive aphasia. *Frontiers in Human Neuroscience*, *10*(NOV2016), 1–8. https://doi.org/10.3389/fnhum.2016.00561

Henry, M. L., Hubbard, H. I., Grasso, S. M., Dial, H. R., Beeson, P. M., Miller, B. L., & Gorno-Tempini, M. L. (2019). Treatment for word retrieval in semantic and logopenic variants of primary progressive aphasia: Immediate and long-term outcomes. *Journal of Speech, Language, and Hearing Research*, *62*(8), 2723–2749. https://doi.org/10.1044/2018_JSLHR-L-18-0144

Beales, A., Bates, K., Cartwright, J., & Whitworth, A. (2019). Lost for words: Perspectives and experiences of people with primary progressive aphasia and Alzheimer's disease and their families of participation in a lexical retrieval intervention. *International Journal of Speech-Language Pathology, 21*(5), 483–492. https://doi.org/10.1080/17549507.2019.1625439

Krajenbrink, T., Croot, K., Taylor-Rubin, C., & Nickels, L. (2020). Treatment for spoken and written word retrieval in the semantic variant of primary progressive aphasia. *Neuropsychological Rehabilitation*, *30*(5), 915–947. https://doi.org/10.1080/09602011.2018.1518780

Lavoie, M., Bier, N., Laforce, R., & Macoir, J. (2020). Improvement in functional vocabulary and generalization to conversation following a self-administered treatment using a smart tablet in primary progressive aphasia. *Neuropsychological Rehabilitation*, *30*(7), 1224–1254. https://doi.org/10.1080/09602011.2019.1570943

Dial, H. R., Hinshelwood, H. A., Grasso, S. M., Hubbard, H. I., Gorno-Tempini, M. L., & Henry, M. L. (2019). Investigating the utility of teletherapy in individuals with primary progressive aphasia. *Clinical Interventions in Aging*, *14*, 453–471. https://doi.org/10.2147/CIA.S178878

Dewar, B. K., Patterson, K., Wilson, B. A., & Graham, K. S. (2009). Re-acquisition of person knowledge in semantic memory disorders. *Neuropsychological Rehabilitation*, *19*(3), 383–421. https://doi.org/10.1080/09602010802278152

Meyer, A. M., Tippett, D. C., Turner, R. S., & Friedman, R. B. (2019). Long-Term maintenance of anomia treatment effects in primary progressive aphasia. *Neuropsychological Rehabilitation*, *29*(9), 1439–1463. https://doi.org/10.1080/09602011.2018.1425146

Croot, K., Raiser, T., Taylor-Rubin, C., Ruggero, L., Ackl, N., Wlasich, E., Danek, A., Scharfenberg, A., Foxe, D., Hodges, J. R., Piguet, O., Kochan, N. A., & Nickels, L. (2019). Lexical retrieval treatment in primary progressive aphasia: An investigation of treatment duration in a heterogeneous case series. *Cortex*, *115*, 133–158. https://doi.org/10.1016/j.cortex.2019.01.009

Cadório, I., Figueiredo, D., Martins, P., Cardoso, R., Santos, J., & Lousada, M. (2021). Combined restorative and compensatory treatment for primary progressive aphasia: a case report. *Aphasiology*, *35*(2), 222–239. https://doi.org/10.1080/02687038.2019.1687842

Kim, M. (2017). Effect of lexical retrieval cascade treatment on naming and discourse of individuals with logopenic variant of primary progressive aphasia(lvPPA). *Clinical Archives of Communication Disorders*, *2*(3), 197–208. https://doi.org/10.21849/cacd.2017.00171

Grasso, S. M., Shuster, K. M., & Henry, M. L. (2019). Comparing the effects of clinician and caregiver-administered lexical retrieval training for progressive anomia. *Neuropsychological Rehabilitation*, *29*(6), 866–895. https://doi.org/10.1080/09602011.2017.1339358

Frattali, C., & Kang, Y. K. (2004). An errorless learning approach to treating dysnomia. *Brain and Language*, *91*(1 SPEC. ISS.), 177–178. https://doi.org/10.1016/j.bandl.2004.06.091

Jokel, R., Rochon, E., & Anderson, N. D. (2010). Errorless learning of computer-generated words in a patient with semantic dementia. *Neuropsychological Rehabilitation*, *20*(1), 16–41. https://doi.org/10.1080/09602010902879859

Savage, S. A., Ballard, K. J., Piguet, O., & Hodges, J. R. (2013). Bringing words back to mind - Improving word production in semantic dementia. *Cortex*, *49*(7), 1823–1832. https://doi.org/10.1016/j.cortex.2012.09.014

Themistocleous, C., Webster, K., & Tsapkini, K. (2021). Effects of tdcs on sound duration in patients with apraxia of speech in primary progressive aphasia. *Brain Sciences*, *11*(3), 1–18. https://doi.org/10.3390/brainsci11030335

Flurie, M., Ungrady, M., & Reilly, J. (2020). Evaluating a maintenance-based treatment approach to preventing lexical dropout in progressive anomia. *Journal of Speech, Language, and Hearing Research*, *63*(12), 4082–4095. https://doi.org/10.1044/2020_JSLHR-20-00059

Rebstock, A. M., & Wallace, S. E. (2020). Effects of a Combined Semantic Feature Analysis and Multimodal Treatment for Primary Progressive Aphasia: Pilot Study. *Communication Disorders Quarterly*, *41*(2), 71–85. https://doi.org/10.1177/1525740118794399

Taylor-Rubin, C., Nickels, L., & Croot, K. (2021). Exploring the effects of verb and noun treatment on verb phrase production in primary progressive aphasia: A series of single case experimental design studies. *Neuropsychological Rehabilitation*, *0*(0), 1–43. https://doi.org/10.1080/09602011.2021.1879174

Beales, A., Whitworth, A., Cartwright, J., Panegyres, P. K., & Kane, R. T. (2021). Making the right connections: Maximizing lexical generalization in lexical impairments in primary progressive aphasia and alzheimer’s disease. *American Journal of Speech-Language Pathology*, *30*(2), 697–712. https://doi.org/10.1044/2020_AJSLP-20-00019

Zhao, Y., Ficek, B., Webster, K., Frangakis, C., Caffo, B., Hillis, A. E., Faria, A., & Tsapkini, K. (2021). White Matter Integrity Predicts Electrical Stimulation (tDCS) and Language Therapy Effects in Primary Progressive Aphasia. *Neurorehabilitation and Neural Repair*, *35*(1), 44–57. https://doi.org/10.1177/1545968320971741

de Aguiar, V., Rofes, A., Wendt, H., Ficek, B. N., Webster, K., & Tsapkini, K. (2021). Treating lexical retrieval using letter fluency and tDCS in primary progressive aphasia: a single-case study. *Aphasiology*, *00*(00), 1–27. https://doi.org/10.1080/02687038.2021.1881432

Paek, E. J., Murray, L. L., & Newman, S. D. (2021). Effects of concurrent action and object naming treatment on naming skills and functional brain activation patterns in primary progressive aphasia: An fMRI study with a case-series design. *Brain and Language*, *218*(November 2020), 104950. https://doi.org/10.1016/j.bandl.2021.104950

Montagut, N., Borrego-Écija, S., Castellví, M., Rico, I., Reñé, R., Balasa, M., Lladó, A., & Sánchez-Valle, R. (2021). Errorless Learning Therapy in Semantic Variant of Primary Progressive Aphasia. *Journal of Alzheimer's Disease*, *79*(1), 415–422. https://doi.org/10.3233/JAD-200904

Mahendra, N., & Tadokoro, A. (2020). Nonfluent Primary Progressive Aphasia: Implications of Palliative Care Principles for Informing Service Delivery. *Topics in Language Disorders*, *40*(3), E7–E24. https://doi.org/10.1097/TLD.0000000000000221

Thompson, C. K., & Shapiro, L. P. (1994). A linguistic-specific approach to treatment of sentence production deficits in aphasia. *Clinical Aphasiology, 22,* 307–323.

Roncero, C., Service, E., De Caro, M., Popov, A., Thiel, A., Probst, S., & Chertkow, H. (2019). Maximizing the Treatment Benefit of tDCS in Neurodegenerative Anomia. *Frontiers in Neuroscience*, *13*(November). https://doi.org/10.3389/fnins.2019.01231

Thompson, C. K., Barbieri, E., Mack, J. E., Wilkins, A., & Xie, K. Y. (2021). Plasticity of sentence processing networks: evidence from a patient with agrammatic variant of primary progressive aphasia (PPA). *Neurocase*, *27*(1), 39–56. https://doi.org/10.1080/13554794.2020.1862241

de Aguiar, V., Zhao, Y., Faria, A., Ficek, B., Webster, K. T., Wendt, H., Wang, Z., Hillis, A. E., Onyike, C. U., Frangakis, C., Caffo, B., & Tsapkini, K. (2020). Brain volumes as predictors of tDCS effects in primary progressive aphasia. *Brain and Language*, *200*(December 2018). https://doi.org/10.1016/j.bandl.2019.104707

Ficek, B. N., Wang, Z., Zhao, Y., Webster, K. T., Desmond, J. E., Hillis, A. E., Frangakis, C., Faria, A. V., Caffo, B., & Tsapkini, K. (2019). Erratum: "The effect of tDCS on functional connectivity in primary progressive aphasia"(NeuroImage: Clinical (2018)19 (703–715), (S2213158218301682), (10.1016/j.nicl.2018.05.023)). *NeuroImage: Clinical*, *22*(March 2019), 101734. https://doi.org/10.1016/j.nicl.2019.101734

Jokel, R., & Anderson, N. D. (2012). Quest for the best: Effects of errorless and active encoding on word re-learning in semantic dementia. *Neuropsychological Rehabilitation*, *22*(2), 187–214. https://doi.org/10.1080/09602011.2011.639626

Schneider, S. L., Thompson, C. K., & Luring, B. (1996). Effects of verbal plus gestural matrix training on sentence production in a patient with primary progressive aphasia. *Aphasiology*, *10*(3), 297–317. https://doi.org/10.1080/02687039608248414

McNeil, M. R., Small, S. L., Masterson, R. J., & Fossett, T. R. D. (1995). Behavioral and Pharmacological Treatment of Lexical-Semantic Deficits in a Single Patient With Primary Progressive Aphasia. *American Journal of Speech-Language Pathology*, *4*(4), 76–87. https://doi.org/10.1044/1058-0360.0404.76

Pattee, C., Von Berg, S., & Ghezzi, P. (2006). Effects of alternative communication on the communicative effectiveness of an individual with a progressive language disorder. *International Journal of Rehabilitation Research*, *29*(2), 151–153. https://doi.org/10.1097/01.mrr.0000210046.02044.4d

Beeson, P. M., King, R. M., Bonakdarpour, B., Henry, M. L., Cho, H., & Rapcsak, S. Z. (2011). Positive effects of language treatment for the logopenic variant of primary progressive aphasia. *Journal of Molecular Neuroscience, 45*(3), 724–736. https://doi.org/10.1007/s12031-011-9579-2

Wong, S. B., Anand, R., Chapman, S. B., Rackley, A., & Zientz, J. (2009). When nouns and verbs degrade: Facilitating communication in semantic dementia. *Aphasiology*, *23*(2), 286–301. https://doi.org/10.1080/02687030801943112

Rapp, B., & Glucroft, B. (2009). The benefits and protective effects of behavioural treatment for dysgraphia in a case of primary progressive aphasia. *Aphasiology*, *23*(2), 236–265. https://doi.org/10.1080/02687030801943054

Louis, M., Espesser, R., Rey, V., Daffaure, V., Di Cristo, A., & Habib, M. (2001). Intensive training of phonological skills in progressive aphasia: A model of brain plasticity in neurodegenerative disease. *Brain and Cognition*, *46*(1–2), 197–201. https://doi.org/10.1016/S0278-2626(01)80065-8

Croot, K., Taylor, C., Abel, S., Jones, K., Krein, L., Hameister, I., Ruggero, L., & Nickels, L. (2015). Measuring gains in connected speech following treatment for word retrieval: a study with two participants with primary progressive aphasia. *Aphasiology*, *29*(11), 1265–1288. https://doi.org/10.1080/02687038.2014.975181

Machado, T. H., Campanha, A. C., Caramelli, P., & Carthery-Goulart, M. T. (2014). Intervençã breve para agramatismo em afasia progressiva primária não fluente: relato de caso. *Dementia e Neuropsychologia*, *8*(3), 291–296. https://doi.org/10.1590/S1980-57642014DN83000014

Dressel, K., Huber, W., Frings, L., Kümmerer, D., Saur, D., Mader, I., Hüll, M., Weiller, C., & Abel, S. (2010). Model-oriented naming therapy in semantic dementia: A single-case fMRI study. *Aphasiology, 24*(12), 1537–1558. https://doi.org/10.1080/02687038.2010.500567

Jokel, R., Meltzer, J., D.R., J., D.M., L., J.C., J., A.N., E., & D.T., C. (2017). Group intervention for individuals with primary progressive aphasia and their spouses: Who comes first? *Journal of Communication Disorders*, *66*, 51–64. https://doi.org/10.1016/j.jcomdis.2017.04.002

Robinson, S., Druks, J., Hodges, J., & Garrard, P. (2009). The treatment of object naming, definition, and object use in semantic dementia: The effectiveness of errorless learning. *Aphasiology*, *23*(6), 749–775. https://doi.org/10.1080/02687030802235195

Meyer, A. M., Tippett, D. C., & Friedman, R. B. (2018). Prophylaxis and remediation of anomia in the semantic and logopenic variants of primary progressive aphasia. *Neuropsychological Rehabilitation*, *28*(3), 352–368. https://doi.org/10.1080/09602011.2016.1148619

Cotelli, M., Manenti, R., Petesi, M., Brambilla, M., Cosseddu, M., Zanetti, O., Miniussi, C., Padovani, A., & Borroni, B. (2014). Treatment of primary progressive aphasias by transcranial direct current stimulation

combined with language training. *Journal of Alzheimer's Disease*, *39*(4), 799–808. https://doi.org/10.3233/JAD-131427

Henry, M. L., Rising, K., DeMarco, A. T., Miller, B. L., Gorno-Tempini, M. L., & Beeson, P. M. (2013). Examining the value of lexical retrieval treatment in primary progressive aphasia: Two positive cases. *Brain and Language*, *127*(2), 145–156. https://doi.org/10.1016/j.bandl.2013.05.018

Henry, M. L., Meese, M. V., Truong, S., Babiak, M. C., Miller, B. L., & Gorno-Tempini, M. L. (2013). Treatment for apraxia of speech in nonfluent variant primary progressive aphasia. *Behavioural Neurology*, *26*(1–2), 77–88. https://doi.org/10.3233/BEN-2012-120260

Henry, M. L., Hubbard, H. I., Grasso, S. M., Mandelli, M. L., Wilson, S. M., Sathishkumar, M. T., Fridriksson, J., Daigle, W., Boxer, A. L., Miller, B. L., & Gorno-Tempini, M. L. (2018). Retraining speech production and fluency in non-fluent/agrammatic primary progressive aphasia. *Brain*, *141*(6), 1799–1814. https://doi.org/10.1093/brain/awy101

Jokel, R., Rochon, E., & Leonard, C. (2006). Treating anomia in semantic dementia: Improvement, maintenance, or both? *Neuropsychological Rehabilitation*, *16*(3), 241–256. https://doi.org/10.1080/09602010500176757

Hameister, I., Nickels, L., Abel, S., & Croot, K. (2017). "Do you have mowing the lawn?"–improvements in word retrieval and grammar following constraint-induced language therapy in primary progressive aphasia. *Aphasiology*, *31*(3), 308–331. https://doi.org/10.1080/02687038.2016.1197558

Meyer, A. M., Getz, H. R., Brennan, D. M., Hu, T. M., & Friedman, R. B. (2016). Telerehabilitation of anomia in primary progressive aphasia. *Aphasiology*, *30*(4), 483–507. https://doi.org/10.1080/02687038.2015.1081142

Heredia, C. G., Sage, K., Lambon Ralph, M. A., & Berthier, M. L. (2009). Relearning and retention of verbal labels in a case of semantic dementia. *Aphasiology*, *23*(2), 192–209. https://doi.org/10.1080/02687030801942999

Meyer, A. M., Snider, S. F., Eckmann, C. B., & Friedman, R. B. (2015). Prophylactic treatments for anomia in the logopenic variant of primary progressive aphasia: cross-language transfer. *Aphasiology*, *29*(9), 1062–1081. https://doi.org/10.1080/02687038.2015.1028327

Murray, L. L. (1998). Longitudinal treatment of primary progressive aphasia: A case study. *Aphasiology*, *12*(7–8), 651–672. https://doi.org/10.1080/02687039808249564

Jokel, R., Cupit, J., Rochon, E., & Leonard, C. (2009). Relearning lost vocabulary in nonfluent progressive aphasia with MossTalk Words®. *Aphasiology*, *23*(2), 175–191. https://doi.org/10.1080/02687030801943005

Farrajota, L., Maruta, C., Maroco, J., Martins, I. P., Guerreiro, M., & de Mendonça, A. (2012). Speech Therapy in Primary Progressive Aphasia: A Pilot Study. *Dementia and Geriatric Cognitive Disorders Extra*, *2*(1), 321–331. https://doi.org/10.1159/000341602

Snowden, J. S., Kindell, J., Thompson, J. C., Richardson, A. M. T., & Neary, D. (2012). Progressive aphasia presenting with deep dyslexia and dysgraphia. *Cortex*, *48*(9), 1234–1239. https://doi.org/10.1016/j.cortex.2012.02.010

Henry, M., Beeson, P., & Rapcsak, S. (2008). Treatment for lexical retrieval in progressive aphasia. *Aphasiology*, *22*(7–8), 826–838. https://doi.org/10.1080/02687030701820055

Routhier, S., Macoir, J., Imbeault, H., Jacques, S., Pigot, H., Giroux, S., Cau, A., & Bier, N. (2014). From smartphone to external semantic memory device: The use of new technologies to compensate for semantic deficits. *Non-Pharmacological Therapies in Different Types of Dementia and Mild Cognitive Impairment: A Wide Perspective from Theory to Practice*, *January*, 93–111.

Newhart, M., Davis, C., Kannan, V., Heidler-Gary, J., Cloutman, L., & Hillis, A. E. (2009). Therapy for naming deficits in two variants of primary progressive aphasia. *Aphasiology*, *23*(7–8), 823–834. https://doi.org/10.1080/02687030802661762

Macoir, J., Leroy, M., Routhier, S., Auclair-Ouellet, N., Houde, M., & Laforce, R. (2015). Improving verb anomia in the semantic variant of primary progressive aphasia: the effectiveness of a semantic-phonological cueing treatment. *Neurocase*, *21*(4), 448–456. https://doi.org/10.1080/13554794.2014.917683

Mayberry, E. J., Sage, K., Ehsan, S., & Lambon Ralph, M. A. (2011). An emergent effect of phonemic cueing following relearning in semantic dementia. *Aphasiology*, *25*(9), 1069–1077. https://doi.org/10.1080/02687038.2011.575203

Hoffman, P., Clarke, N., Jones, R. W., & Noonan, K. A. (2015). Vocabulary relearning in semantic dementia: Positive and negative consequences of increasing variability in the learning experience. *Neuropsychologia*, *76*, 240–253. https://doi.org/10.1016/j.neuropsychologia.2015.01.015

Flanagan, K. J., Copland, D. A., Van Hees, S., Byrne, G. J., & Angwin, A. J. (2016). Semantic feature training for the treatment of anomia in alzheimer disease: A preliminary investigation. *Cognitive and Behavioral Neurology*, *29*(1), 32–43. https://doi.org/10.1097/WNN.0000000000000088

Cress, C. J., & King, J. M. (1999). AAC strategies for people with primary progressive aphasia without dementia: Two case studies. *AAC: Augmentative and Alternative Communication*, *15*(4), 248–259. https://doi.org/10.1080/07434619912331278785

Burdea, G. C., Polistico, K., House, G. P., Liu, R. R., Muñiz, R., Macaro, N. A., & Slater, L. M. (2015). Novel integrative virtual rehabilitation reduces symptomatology of primary progressive aphasia - A case report. *International Journal of Neuroscience*, *125*(12), 949–958. https://doi.org/10.3109/00207454.2014.993392

Cartwright, J., & Elliott, K. A. E. (2009). Promoting strategic television viewing in the context of progressive language impairment. *Aphasiology*, *23*(2), 266–285. https://doi.org/10.1080/02687030801942932

Cotelli, M., Manenti, R., Paternicò, D., Cosseddu, M., Brambilla, M., Petesi, M., Premi, E., Gasparotti, R., Zanetti, O., Padovani, A., & Borroni, B. (2016). Grey Matter Density Predicts the Improvement of Naming Abilities After tDCS Intervention in Agrammatic Variant of Primary Progressive Aphasia. *Brain Topography*, *29*(5), 738–751. https://doi.org/10.1007/s10548-016-0494-2

Andrade-Calderón, P., Salvador-Cruz, J., & Sosa-Ortiz, A. L. (2015). Positive impact of speech therapy in progressive non-fluent aphasia. [Impacto positivo da terapia da linguagem em afasia progressiva não fluente; Impacto positivo de la terapia del lenguaje en afasia progresiva no fluente]. *Acta Colombiana de Psicologia*, *18*(2), 101–114. https://doi.org/10.14718/ACP.2015.18.2.9

Tsapkini, K., Webster, K. T., Ficek, B. N., Desmond, J. E., Onyike, C. U., Rapp, B., Frangakis, C. E., & Hillis, A. E. (2018). Electrical brain stimulation in different variants of primary progressive aphasia: A

randomized clinical trial. *Alzheimer's and Dementia: Translational Research and Clinical Interventions*, *4*, 461–472. https://doi.org/10.1016/j.trci.2018.08.002

Bier, N., Brambati, S., Macoir, J., Paquette, G., Schmitz, X., Belleville, S., Faucher, C., & Joubert, S. (2015). Relying on procedural memory to enhance independence in daily living activities: Smartphone use in a case of semantic dementia. *Neuropsychological Rehabilitation*, *25*(6), 913–935. https://doi.org/10.1080/09602011.2014.997745

Bier, N., MacOir, J., Joubert, S., Bottari, C., Chayer, C., Pigot, H., Giroux, S., & Team, S. (2011). Cooking Shrimp á la Créole: A pilot study of an ecological rehabilitation in semantic dementia. *Neuropsychological Rehabilitation*, *21*(4), 455–483. https://doi.org/10.1080/09602011.2011.580614

Mooney, A., Bedrick, S., Noethe, G., Spaulding, S., & Fried-Oken, M. (2018). Mobile technology to support lexical retrieval during activity retell in primary progressive aphasia. *Aphasiology*, *32*(6), 666–692. https://doi.org/10.1080/02687038.2018.1447640

Mooney, A., Beale, N., & Fried-Oken, M. (2018). Group Communication Treatment for Individuals with PPA and Their Partners. *Seminars in Speech and Language*, *39*(3), 257–269. https://doi.org/10.1055/s-0038-1660784

Roncero, C., Kniefel, H., Service, E., Thiel, A., Probst, S., & Chertkow, H. (2017). Inferior parietal transcranial direct current stimulation with training improves cognition in anomic Alzheimer's disease and frontotemporal dementia. *Alzheimer's and Dementia: Translational Research and Clinical Interventions*, *3*(2), 247–253. https://doi.org/10.1016/j.trci.2017.03.003

Meyer, A. M., Faria, A. V., Tippett, D. C., Hillis, A. E., & Friedman, R. B. (2017). The relationship between baseline volume in temporal areas and post-treatment naming accuracy in primary progressive aphasia. *Aphasiology*, *31*(9), 1059–1077. https://doi.org/10.1080/02687038.2017.1296557

Jafari, S., Khatoonabadi, A. R., Noroozian, M., Mehri, A., Ashayeri, H., & Nickels, L. (2018). The Effect of Word Retrieval Therapy in Primary Progressive Aphasia: A Single-Case Study. *Archives of Neuroscience*, *In Press*(In Press). https://doi.org/10.5812/ans.67577

Kindell, J., Wilkinson, R., Sage, K., & Keady, J. (2018). Combining music and life story to enhance participation in family interaction in semantic dementia: a longitudinal study of one family's experience. *Arts and Health*, *10*(2), 165–180. https://doi.org/10.1080/17533015.2017.1342269

Harris, A. D., Wang, Z., Ficek, B., Webster, K., Edden, R. A., & Tsapkini, K. (2019). Reductions in GABA following a tDCS-language intervention for primary progressive aphasia. *Neurobiology of Aging*, *79*, 75–82. https://doi.org/10.1016/j.neurobiolaging.2019.03.011

Fenner, A. S., Webster, K. T., Ficek, B. N., Frangakis, C. E., & Tsapkini, K. (2019). Written verb naming improves after tDCS over the left IFG in primary progressive aphasia. *Frontiers in Psychology*, *10*(JUN), 1–13. https://doi.org/10.3389/fpsyg.2019.01396

Villanelli, F., Russo, A., Nemni, R., & Farina, E. (2014). Effectiveness (or Not?) of cognitive rehabilitation in a person with Semantic Dementia. *Non-Pharmacological Therapies in Different Types of Dementia and Mild Cognitive Impairment: A Wide Perspective from Theory to Practice*, *January*, 113–119.

Senaha, M. L. H., Brucki, S. M. D., & Nitrini, R. (2010). Reabilitação na demência semântica: Estudo da eficácia da reaquisição lexical em três pacientes. *Dementia e Neuropsychologia*, *4*(4), 306–312. https://doi.org/10.1590/S1980-57642010DN40400009

Suárez-González, A., Heredia, C. G., Savage, S. A., Gil-Néciga, E., García-Casares, N., Franco-Macías, E., Berthier, M. L., & Caine, D. (2015). Restoration of conceptual knowledge in a case of semantic dementia. *Neurocase*, *21*(3), 309–321. https://doi.org/10.1080/13554794.2014.892624

Tsapkini, K., & Hillis, A. E. (2013). Spelling intervention in post-stroke aphasia and primary progressive aphasia. *Behavioural Neurology*, *26*(1–2), 55–66. https://doi.org/10.3233/BEN-2012-110240

Rogalski, Y., & Edmonds, L. (2008). Attentive reading and constrained summarisation (ARCS) treatment in primary progressive aphasia: A case study. *Aphasiology*, *22*(7–8), 763–775. https://doi.org/10.1080/02687030701803796

Schaffer, K. M., Wauters, L., Berstis, K., Grasso, S. M., & Henry, M. L. (2020). Modified script training for nonfluent/agrammatic primary progressive aphasia with significant hearing loss: A single-case experimental design. *Neuropsychological Rehabilitation*, 1–30. https://doi.org/10.1080/09602011.2020.1822188

Whitworth, A., Cartwright, J., Beales, A., Leitão, S., Panegyres, P. K., & Kane, R. (2018). Taking words to a new level: a preliminary investigation of discourse intervention in primary progressive aphasia. In *Aphasiology* (Vol. 32, Issue 11, pp. 1284–1309). Routledge. https://doi.org/10.1080/02687038.2017.1390543

Hung, J., Bauer, A., Grossman, M., Hamilton, R. H., Coslett, H. B., & Reilly, J. (2017). Semantic feature training in combination with transcranial direct current stimulation (tDCS) for progressive anomia. *Frontiers in Human Neuroscience*, *11*, 253. https://doi.org/10.3389/fnhum.2017.00253

Savage, S. A., Piguet, O., & Hodges, J. R. (2015). Cognitive intervention in semantic dementia maintaining words over time. *Alzheimer Disease and Associated Disorders*, *29*(1), 55–62. https://doi.org/10.1097/WAD.0000000000000053

Savage, S. A., Piguet, O., & Hodges, J. R. (2014). Giving words new life: Generalization of word retraining outcomes in semantic dementia. *Journal of Alzheimer's Disease*, *40*(2), 309–317. https://doi.org/10.3233/JAD-131826

Beales, A., Cartwright, J., Whitworth, A., & Panegyres, P. K. (2016). Exploring generalisation processes following lexical retrieval intervention in primary progressive aphasia. *International Journal of Speech-Language Pathology*, *18*(3), 299–314. https://doi.org/10.3109/17549507.2016.1151936

Rogalski, E. J., Saxon, M., McKenna, H., Wieneke, C., Rademaker, A., Corden, M. E., Borio, K., Mesulam, M. M., & Khayum, B. (2016). Communication Bridge: A pilot feasibility study of Internet-based speech-language therapy for individuals with progressive aphasia. *Alzheimer's and Dementia: Translational Research and Clinical Interventions*, *2*(4), 213–221. https://doi.org/10.1016/j.trci.2016.08.005

Reilly, J. (2016). How to constrain and maintain a lexicon for the treatment of progressive semantic naming deficits: Principles of item selection for formal semantic therapy. *Neuropsychological Rehabilitation*, *26*(1), 126–156. https://doi.org/10.1080/09602011.2014.1003947

Snowden, J. S., & Neary, D. (2002). Relearning of verbal labels in semantic dementia. *Neuropsychologia*, *40*(10), 1715–1728. https://doi.org/10.1016/S0028-3932(02)00031-3

Jokel, R., Kielar, A., Anderson, N. D., Black, S. E., Rochon, E., Graham, S., Freedman, M., & Tang-Wai, D. F. (2016). Behavioural and neuroimaging changes after naming therapy for semantic variant primary

progressive aphasia. *Neuropsychologia*, *89,* 191–216. https://doi.org/10.1016/j.neuropsychologia.2016.06.009

Tsapkini, K., Frangakis, C., Gomez, Y., Davis, C., & Hillis, A. E. (2014). Augmentation of spelling therapy with transcranial direct current stimulation in primary progressive aphasia: Preliminary results and challenges. *Aphasiology*, *28*(8–9), 1112–1130. https://doi.org/10.1080/02687038.2014.930410

Mayberry, E. J., Sage, K., Ehsan, S., & Lambon Ralph, M. A. (2011). Relearning in semantic dementia reflects contributions from both medial temporal lobe episodic and degraded neocortical semantic systems: Evidence in support of the complementary learning systems theory. *Neuropsychologia*, *49*(13), 3591–3598. https://doi.org/10.1016/j.neuropsychologia.2011.09.010

Graham, K. S., Patterson, K., Pratt, K. H., & Hodges, J. R. (1999). Relearning and subsequent forgetting of semantic category exemplars in a case of semantic dementia. *Neuropsychology*, *13*(3), 359–380. https://doi.org/10.1037/0894-4105.13.3.359

Bier, N., Macoir, J., Gagnon, L., Van der Linden, M., Louveaux, S., & Desrosiers, J. (2009). Known, lost, and recovered: Efficacy of formal-semantic therapy and spaced retrieval method in a case of semantic dementia. *Aphasiology*, *23*(2), 210–235. https://doi.org/10.1080/00207590801942906

Marcotte, K., & Ansaldo, A. I. (2010). The neural correlates of semantic feature analysis in chronic aphasia: Discordant patterns according to the etiology. *Seminars in Speech and Language*, *31*(1), 52–63. https://doi.org/10.1055/s-0029-1244953

Graham, K. S., Patterson, K., Pratt, K. H., & Hodges, J. R. (2001). Can repeated exposure to “forgotten” vocabulary help alleviate word-finding difficulties in semantic dementia? An illustrative case study. *Neuropsychological Rehabilitation*, *11*(3–4), 429–454. https://doi.org/10.1080/09602010042000060
