# Supplementary material for: Behavioral Treatment for Speech and Language in Primary Progressive Aphasia and Primary Progressive Apraxia of Speech: A Systematic Review
Source: Neuropsychol Rev. 2023 Oct 4;34(3):882–923. doi: 10.1007/s11065-023-09607-1 (PMC11473583; doi:10.1007/s11065-023-09607-1)
Supplement: Supplementary file 6 — Supplementary file6 (PDF 128 KB) [file 11065_2023_9607_MOESM6_ESM.pdf]

Wauters, L.D., Croot, K., Dial, H.R., Duffy, J.R., Grasso, S.M., Kim, E., Schaffer, K.M., Ballard, K.J., Clark, H.M., Kohley, L., Murray, L.L., Rogalski, E.J., Figeys, M., Milman, L., Henry, M.L., Behavioral treatment for speech and language in primary progressive aphasia and primary progressive apraxia of speech: A systematic review. *Neuropsychology Review*.

Corresponding author: Maya Henry, Department of Speech, Language, and Hearing Sciences, The University of Texas at Austin, 2504A Whitis Ave. (A1100), Austin, TX 78712-0114, E-mail: maya.henry@austin.utexas.edu.

Supplementary Materials 6: *Ratings of diagnostic evidence for PPA and AOS for all studies*

**Study reference**	**PPA Diagnostic Evidence Rating**	**AOS Diagnostic Evidence Rating**	**Study reference**	**PPA Diagnostic Evidence Rating**	**AOS Diagnostic Evidence Rating**
Andrade-Calderón et al. (2015)	1	N/A	Lavoie et al. (2019)	1	N/A
Beales et al. (2016)	2	N/A	Louis et al. (2001)	3	N/A
Beales et al. (2019)	4	N/A	Machado et al. (2014)	1	5
Beales et al. (2021)	3	N/A	Macoir et al. (2015)	1	N/A
Beeson et al. (2011)	1	N/A	Mahendra & Tadokoro (2020)	1	3
Bier et al. (2009)	1	N/A	Marcotte et al. (2010)	4	N/A
Bier et al. (2011)	1	N/A	Mayberry et al. (2011a)	1	N/A
Bier et al. (2015)	1	N/A	Mayberry et al. (2011b)	1	N/A
Burdea et al. (2015)	3	N/A	Mcneil et al. (1995)	2	N/A
Cadorio et al. (2019)	2	N/A	Meyer et al. (2015)	1	N/A
Cartwright & Elliott (2009)	3	N/A	Meyer et al. (2017)	1	N/A
Cotelli et al. (2014)	3	N/A	Meyer et al. (2019)	1	N/A
Cotelli et al. (2016)	3	N/A	Meyer, Getz, et al. (2016)	1	N/A
Cress & King (1999)	3	N/A	Meyer, Tippett, et al. (2016)	1	N/A
Croot et al. (2015)	P1=5; P2=3	4	Montagut et al. (2021)	2	N/A
Croot et al. (2019)	2	N/A	Mooney, Beales, et al. (2018)	4	N/A
de Aguiar et al. (2019)	1	N/A	Mooney, Bedrick, et al. (2018)	1 for 4 P’s; 3 for 2 P’s	N/A

de Aguiar et al. (2020)	4	N/A	Murray (1998)	2	4
de Aguiar et al. (2021)	2	N/A	Newhart et al. (2009)	1	N/A
Dewar et al. (2009)	2	N/A	Paek et al. (2021)	3	N/A
Dial et al. (2019)	3	N/A	Pattee et al. (2006)	3	4
Dressel et al. (2010)	1	N/A	Rapp & Glucroft (2009)	2	N/A
Evans et al. (2016)	2	N/A	Rebstock & Wallace (2020)	3	N/A
Farrajota et al. (2012)	2	N/A	Reilly (2016)	2	N/A
Fenner et al. (2019)	2	N/A	Robinson et al. (2009)	2	N/A
Ficek et al. (2018)	2	N/A	Rogalski & Edmonds (2008)	3	N/A
Flanagan et al. (2016)	4	N/A	Rogalski et al. (2016)	4	N/A
Flurie et al. (2020)	2	N/A	Roncero et al. (2017)	3	N/A
Frattali (2004)	1	N/A	Roncero et al. (2019)	5	N/A
Graham et al. (1999)	2	N/A	Routhier et al. (2011)	1	N/A
Graham et al. (2001)	2	N/A	Savage et al. (2013)	1	N/A
Grasso et al. (2019)	1	N/A	Savage et al. (2014)	1	N/A
Hameister et al. (2016)	1	N/A	Savage et al. (2015)	1	N/A
Harris et al. (2019)	2	N/A	Schaffer et al. (2020)	1	4
Henry et al. (2008)	3	4	Schneider et al. (1996)	1	N/A
Henry et al. (2018)	2	4	Senaha et al. (2010)	2	N/A
Henry et al. (2019)	1	N/A	Snowden & Neary (2002)	1	N/A
Henry, Meese, et al. (2013)	1	1	Snowden et al. (2012)	1	N/A
Henry, Rising, et al. (2013)	1	N/A	Suarez-Gonzalez et al. (2015)	1	N/A
Heredia et al. (2009)	1	N/A	Suárez-González et al. (2016)	1	N/A
Hoffman et al. (2015)	2	N/A	Taylor-Rubin et al. (2021)	1	N/A
Hung et al. (2017)	3	N/A	Themistocleous et al. (2021)	1	4
Jafari et al. (2018)	2	5	Thompson & Shapiro (1994)	3	N/A

Jokel & Anderson (2012)	2	N/A	Thompson et al. (2020)	1	N/A
Jokel et al. (2006)	1	N/A	Tsapkini & Hillis (2013)	2	N/A
Jokel et al. (2009)	2	N/A	Tsapkini et al. (2014)	3	N/A
Jokel et al. (2010)	1	N/A	Tsapkini et al. (2018)	2	N/A
Jokel et al. (2016)	1	N/A	Villanelli et al. (2011)	2	N/A
Jokel et al. (2017)	3	4	Whitworth et al. (2017)	1	N/A
Kim (2017)	P1=1; P2=2	N/A	Wong et al. (2009)	1	N/A
Kindell et al. (2018)	5	N/A	Zhao et al. (2021)	4	N/A
Krajenbrink et al. (2018)	2	N/A			
