# Supplementary material for: Behavioral Treatment for Speech and Language in Primary Progressive Aphasia and Primary Progressive Apraxia of Speech: A Systematic Review
Source: Neuropsychol Rev. 2023 Oct 4;34(3):882–923. doi: 10.1007/s11065-023-09607-1 (PMC11473583; doi:10.1007/s11065-023-09607-1)
Supplement: Supplementary file 7 — Supplementary file7 (PDF 194 KB) [file 11065_2023_9607_MOESM7_ESM.pdf]

Wauters, L.D., Croot, K., Dial, H.R., Duffy, J.R., Grasso, S.M., Kim, E., Schaffer, K.M., Ballard, K.J., Clark, H.M., Kohley, L., Murray, L.L., Rogalski, E.J., Figeys, M., Milman, L., Henry, M.L., Behavioral treatment for speech and language in primary progressive aphasia and primary progressive apraxia of speech: A systematic review**.** *Neuropsychology Review*.

Corresponding author: Maya Henry, Department of Speech, Language, and Hearing Sciences, The University of Texas at Austin, 2504A Whitis Ave. (A1100), Austin, TX 78712-0114, E-mail: maya.henry@austin.utexas.edu.

Supplementary Materials 7: *Appraisal Point System (APS) scores* (Oren et al., 2014) *for all studies*

**Study**	**APS #1**	**APS #2**	**APS #3**	**APS #4**	**APS #5**	**APS #6**	**APS #7**	**APS #8**	**APS #9**	**APS Total**
Andrade-Calderón et al. (2015)	1	0	0	1	0	0	0	1	0	3
Beales et al. (2016)	1	0	0	1	1	0	1	1	1	8
Beales et al. (2019)	1	0	0	0	0	0	0	1	1	3
Beales et al. (2021)	1	1	0	0	1	0	1	1	1	9
Beeson et al. (2011)	1	0	0	1	1	0	1	1	1	8
Bier et al. (2009)	1	1	0	1	1	0	1	0	1	9
Bier et al. (2011)	1	0	0	1	1	0	1	1	1	8
Bier et al. (2015)	1	0	0	1	1	0	1	1	1	8
Burdea et al. (2015)	0	0	0	1	0	0	0	1	0	2
Cadorio et al. (2019)	1	0	0	0	1	0	1	1	1	7
Cartwright & Elliott (2009)	1	0	0	0	1	0	0	0	1	4
Cotelli et al. (2014)	1	1	0	1	1	0	1	1	1	10
Cotelli et al. (2016)	1	0	0	1	1	0	1	1	1	8
Cress & King (1999)	0	0	0	1	0	0	0	1	1	3
Croot et al. (2015)	1	1	0	1	1	0	1	1	1	10
Croot et al. (2019)	1	1	0	1	1	0	1	1	1	10
de Aguiar et al. (2019)	1	0	0	1	1	0	1	1	1	8
de Aguiar et al. (2020)	1	0	0	0	1	0	0	1	1	5
de Aguiar et al. (2021)	1	0	0	1	1	0	1	1	1	8
Dewar et al. (2009)	1	0	0	1	1	0	1	1	1	8
Dial et al. (2019)	1	1	0	1	1	0	1	1	1	10
Dressel et al. (2010)	1	0	0	1	1	0	1	1	1	8
Evans et al. (2016)	1	0	0	1	1	0	1	1	1	8
Farrajota et al. (2012)	1	1	0	1	1	0	1	0	1	9
Fenner et al. (2019)	1	0	0	1	0	0	1	1	1	6
Ficek et al. (2018)	1	0	0	0	1	0	1	0	1	6
Flanagan et al. (2016)	1	0	0	0	1	0	1	0	1	6
Flurie et al. (2020)	1	0	0	1	1	0	1	1	1	8
Frattali (2004)	1	1	0	1	1	0	0	1	1	8
Graham et al. (1999)	1	0	0	1	1	0	1	1	1	8
Graham et al. (2001)	1	0	0	1	1	0	1	1	1	8
Grasso et al. (2019)	1	1	0	1	1	1	1	1	1	12
Hameister et al. (2016)	1	1	0	1	1	0	1	1	1	10
Harris et al. (2019)	1	0	0	1	0	0	1	0	1	5
Henry et al. (2008)	1	1	0	1	1	0	1	1	1	10
Henry et al. (2018)	1	1	0	1	1	1	1	1	1	12

Henry et al. (2019)	1	1	0	1	1	0	1	1	1	10
Henry, Meese, et al. (2013)	1	0	0	1	1	0	1	1	1	8
Henry, Rising, et al. (2013)	1	1	0	1	1	0	1	1	1	10
Heredia et al. (2009)	1	1	0	1	1	0	1	1	1	10
Hoffman et al. (2015)	1	0	0	1	1	0	0	1	1	6
Hung et al. (2017)	1	1	0	1	1	0	1	1	1	10
Jafari et al. (2018)	1	0	0	1	1	0	1	1	0	7
Jokel & Anderson (2012)	1	1	0	1	1	0	1	1	1	10
Jokel et al. (2006)	1	0	0	1	1	0	1	1	1	8
Jokel et al. (2009)	1	1	0	1	1	0	1	1	1	10
Jokel et al. (2010)	1	1	0	1	1	0	1	1	1	10
Jokel et al. (2016)	1	0	0	1	1	0	1	0	1	7
Jokel et al. (2017)	1	1	0	0	1	0	1	1	1	9
Kim (2017)	1	1	0	1	1	0	1	1	1	10
Kindell et al. (2018)	1	0	0	0	0	0	0	1	1	3
Krajenbrink et al. (2018)	1	1	0	1	1	0	1	0	1	9
Lavoie et al. (2019)	1	1	0	1	1	1	1	1	1	12
Louis et al. (2001)	0	0	0	0	0	0	0	0	1	1
Machado et al. (2014)	1	0	0	1	0	0	1	1	1	6
Macoir et al. (2015)	1	1	0	1	1	0	1	1	1	10
Mahendra & Tadokoro (2020)	1	0	0	1	0	0	0	1	1	4
Marcotte et al. (2010)	1	0	0	1	1	0	0	0	1	5
Mayberry et al. (2011a)	1	0	0	1	1	0	1	0	1	7
Mayberry et al. (2011b)	1	0	0	1	1	0	0	1	1	6
Mcneil et al. (1995)	1	1	0	1	1	0	0	1	1	8
Meyer et al. (2015)	1	0	0	1	1	0	0	0	1	5
Meyer et al. (2017)	1	1	0	1	1	0	0	0	1	7
Meyer et al. (2019)	1	1	0	1	1	0	0	1	1	8
Meyer, Getz, et al. (2016)	1	0	0	1	1	0	0	1	1	6
Meyer, Tippett, et al. (2016)	1	1	0	1	1	0	0	0	1	7
Montagut et al. (2021)	1	0	0	1	1	0	0	0	1	5
Mooney, Beales, et al. (2018)	1	0	0	0	0	1	0	1	1	5
Mooney, Bedrick, et al. (2018)	1	0	0	0	0	1	0	1	0	4
Murray (1998)	1	0	0	1	0	0	0	1	1	4
Newhart et al. (2009)	1	0	0	1	1	0	1	1	1	8
Paek et al. (2021)	1	0	0	1	1	0	1	1	1	8
Pattee et al. (2006)	1	0	0	0	1	0	0	0	1	4
Rapp & Glucroft (2009)	1	0	0	1	1	0	1	0	1	7
Rebstock & Wallace (2020)	1	0	0	1	1	0	1	0	1	7
Reilly (2016)	1	0	0	1	1	0	1	0	1	7
Robinson et al. (2009)	1	0	0	1	1	0	1	1	1	8
Rogalski & Edmonds (2008)	1	0	0	1	1	0	0	1	1	6
Rogalski et al. (2016)	0	0	0	0	1	0	0	1	0	3
Roncero et al. (2017)	0	0	0	0	1	0	1	1	1	6
Roncero et al. (2019)	1	1	0	0	1	0	1	1	1	9
Routhier et al. (2011)	1	0	0	1	0	0	0	1	1	4
Savage et al. (2013)	1	1	0	1	1	0	1	1	1	10
Savage et al. (2014)	1	0	0	1	1	0	1	1	1	8
Savage et al. (2015)	1	0	0	1	1	0	1	1	1	8
Schaffer et al. (2020)	1	1	0	1	1	1	1	1	1	12

Schneider et al. (1996)	1	0	0	0	1	1	0	1	1	7
Senaha et al. (2010)	1	0	0	0	1	0	0	0	1	4
Snowden & Neary (2002)	1	0	0	1	1	0	1	0	1	7
Snowden et al. (2012)	1	0	0	1	1	0	0	1	1	6
Suarez-Gonzalez et al. (2015)	1	1	0	1	0	0	1	1	1	8
Suárez-González et al. (2016)	1	0	0	1	1	0	1	1	1	8
Taylor-Rubin et al. (2021)	1	1	0	1	1	0	1	1	1	10
Themistocleous et al. (2021)	1	0	0	0	1	0	0	1	1	5
Thompson & Shapiro (1994)	1	1	0	0	1	0	0	0	1	6
Thompson et al. (2020)	1	1	0	1	0	0	1	1	1	8
Tsapkini & Hillis (2013)	1	0	0	1	1	0	0	0	1	5
Tsapkini et al. (2014)	1	0	0	0	1	0	1	1	1	7
Tsapkini et al. (2018)	1	0	0	1	0	0	1	1	1	6
Villanelli et al. (2011)	0	0	0	1	1	0	0	1	0	4
Whitworth et al. (2017)	1	0	0	1	1	0	1	1	1	8
Wong et al. (2009)	0	0	0	1	0	0	0	1	1	3
Zhao et al. (2021)	0	1	0	1	1	0	1	1	1	9

Notes: Item #1: Rationale – Focused and complete PICO, Unbiased review of the literature; Item #2: Comparison/Control (2 points) – Appropriate comparison, including between-subject designs and within-subject designs, counterbalancing stimuli, or pre- and post-testing group; Item #3 (2 points) – Participants randomly assigned to treatment conditions; Item #4 – Participants: All three adequately described – Diagnosis, Severity, Demographics; Item #5 – Measures & Materials (2 points): Rationale described for selection of outcome measures, Clearly define how "goal mastery" will be measured; Item #6 – Procedures (2 points): Study procedures are well articulated and implemented as described (in order to receive this point, studies had to report a measure of adherence or fidelity); Item #7 – Statistical reporting (2 points): 2 of 3: Appropriate use of statistical procedures, Provides descriptive statistics, and effect sizes reported; Item #8 – Clinical Significance: 2 of 4: report generalization, maintenance post-treatment, study carried out in real-world settings, use of functional stimuli; Item #9 – Discussion/Interpretation: Linked to PICO, Appropriate conclusion in relation to study design and results, unexpected findings addressed and alternative explanations offered, study limitations acknowledged.

Notes on Consistency of Ratings: Items on the APS that were subject to higher rates of initial disagreement between raters (i.e., disagreement rate of 20% or above) were related to the adequate reporting of participant information (APS #4), the adequate reporting of procedures (APS #6), the demonstration of adequate experimental control (APS #2), and the demonstration of generalization/external validity (APS #8). Some of the higher rates of discrepancy were due to ambiguous wording of scale items, which was clarified during consensus meetings. As noted, two study authors (LW and MH) reviewed consensus ratings for quality rating scales to ensure that ratings were consistently applied, with special attention to APS items #2 and #6 due to the ambiguous wording of these items.
