# Supplementary material for: Behavioral Treatment for Speech and Language in Primary Progressive Aphasia and Primary Progressive Apraxia of Speech: A Systematic Review
Source: Neuropsychol Rev. 2023 Oct 4;34(3):882–923. doi: 10.1007/s11065-023-09607-1 (PMC11473583; doi:10.1007/s11065-023-09607-1)
Supplement: Supplementary file 8 — Supplementary file8 (PDF 4.8 KB) [file 11065_2023_9607_MOESM8_ESM.pdf]

Wauters, L.D., Croot, K., Dial, H.R., Duffy, J.R., Grasso, S.M., Kim, E., Schaffer, K.M., Ballard, K.J., Clark, H.M., Kohley, L., Murray, L.L., Rogalski, E.J., Figeys, M., Milman, L., Henry, M.L., Behavioral treatment for speech and language in primary progressive aphasia and primary progressive apraxia of speech: A systematic review**.** *Neuropsychology Review*.

Corresponding author: Maya Henry, Department of Speech, Language, and Hearing Sciences, The University of Texas at Austin, 2504A Whitis Ave. (A1100), Austin, TX 78712-0114, E-mail: maya.henry@austin.utexas.edu.

Supplementary Materials 8: *Single-Case Experimental Design (SCED) Scale* (Tate et al., 2008, 2015) *scores for multiple baseline, reversal/withdraw, alternating treatment, and changing criterion designs*

**Study**	**SCED #1***	**SCED #2**	**SCED #3**	**SCED #4**	**SCED #5**	**SCED #6**	**SCED #7**	**SCED #8**	**SCED #9**	**SCED #10**	**SCED #11**	**SCED Total**
Beeson et al. (2011)	1	1	1	1	1	0	0	0	1	0	1	6
Bier et al. (2015)	1	1	0	0	1	1	0	0	1	0	1	5
Bier et al. (2009)	1	1	0	1	1	1	0	0	1	0	0	5
Bier et al. (2011)	1	1	0	0	1	1	0	0	1	0	1	5
Frattali (2004)	1	1	1	1	1	1	0	0	1	0	1	7
Henry et al. (2008)	1	1	1	0	1	1	0	0	1	1	0	6
Henry, Rising, et al. (2013)	1	1	1	1	1	1	0	0	1	1	1	8
Jokel et al. (2009)	1	1	1	1	1	1	0	0	1	1	1	8
Jokel et al. (2010)	1	1	1	1	1	1	0	0	1	0	1	7
Macoir et al. (2015)	1	1	1	1	1	1	1	0	1	0	1	8
Mcneil et al. (1995)	1	1	1	1	1	1	0	1	0	0	0	6
Mooney, Bedrick, et al. (2018)	1	1	0	0	0	0	1	0	0	1	0	3
Routhier et al. (2011)	1	0	0	1	1	0	0	0	0	0	0	2
Savage et al. (2013)	1	1	1	1	1	1	0	0	1	1	0	7
Schneider et al. (1996)	0	1	0	0	1	1	1	1	0	0	0	5
Kim (2017)	1	1	1	0	1	1	0	0	0	0	0	4
Lavoie et al. (2019)	1	1	1	1	1	1	0	0	1	1	1	8
Paek et al. (2021)	1	1	0	1	1	1	0	0	1	1	1	7

Rebstock & Wallace (2020)	0	1	0	1	1	1	1	0	1	0	1	7
Schaffer et al. (2020)	1	1	1	0	1	1	1	0	1	0	1	7
Thompson & Shapiro (1994)	0	1	1	1	1	1	1	0	0	0	1	7
Thompson et al. (2020)	1	1	1	0	1	1	0	0	1	0	1	6

Notes: Item #1 is not included in the overall quality score (out of 10). Item #1: Clinical history was specified. Item #2: Target behaviors. Precise and repeatable measures that are operationally defined. Item #3: Study design shows cause and effect; Item #4: Sampling of behavior at baseline (minimum of three measurements); Item #5: Sampling behavior during treatment; Item #6: Raw data record; Item #7: Inter-rater reliability; Item #8: Independence of Assessors; Item #9: Comparison with statistical analysis; Item #10: Demonstration of replication; Item #10: Demonstration of functional utility.

Notes on Consistency of Ratings: Items on the SCED that were subject to higher rates of initial disagreement between raters (i.e., disagreement rate of 20% or above) were related to the adequate reporting of participant information (SCED #1), the demonstration of adequate experimental design (SCED #5), and the demonstration of generalization/external validity (SCED #11). Some of the higher rates of discrepancy were due to ambiguous wording of scale items, which was clarified during consensus meetings.
