# Supplementary material for: Behavioral Treatment for Speech and Language in Primary Progressive Aphasia and Primary Progressive Apraxia of Speech: A Systematic Review
Source: Neuropsychol Rev. 2023 Oct 4;34(3):882–923. doi: 10.1007/s11065-023-09607-1 (PMC11473583; doi:10.1007/s11065-023-09607-1)
Supplement: Supplementary file 9 — Supplementary file9 (PDF 75.9 KB) [file 11065_2023_9607_MOESM9_ESM.pdf]

Wauters, L.D., Croot, K., Dial, H.R., Duffy, J.R., Grasso, S.M., Kim, E., Schaffer, K.M., Ballard, K.J., Clark, H.M., Kohley, L., Murray, L.L., Rogalski, E.J., Figeys, M., Milman, L., Henry, M.L., Behavioral treatment for speech and language in primary progressive aphasia and primary progressive apraxia of speech: A systematic review**.**
Neuropsychology Review.

Corresponding author: Maya Henry, Department of Speech, Language, and Hearing Sciences, The University of Texas at Austin, 2504A Whitis Ave. (A1100), Austin, TX 78712-0114, E-mail: maya.henry@austin.utexas.edu.

Supplementary Materials 9: *PEDro-P Scale scores* (Murray et al., 2013) *for non-randomized controlled trials*

**Study**	**PEDro #1***	**PEDro #2**	**PEDro #3**	**PEDro #4**	**PEDro #5**	**PEDro #6**	**PEDro #7**	**PEDro #8**	**PEDro #9**	**PEDro #10**	**PEDro #11**	**PEDro Total**
Dial et al. (2019)	1	0	0	1	0	0	0	1	1	1	1	5
Farrajota et al. (2012)	1	0	0	1	0	0	0	1	1	1	1	5
Jokel et al. (2017)	1	0	0	0	0	0	0	1	1	1	0	3

Notes: *Item #1 relates to external validity and so is not counted in the final quality rating score (out of 10). Item #1: Eligibility criteria were specified; Item #2: Subjects were randomly allocated to interventions (in a crossover study, subjects were randomly allocated an order in which treatments were received); Item #3: Allocation was concealed; Item #4: The intervention groups were similar at baseline regarding the most important prognostic indicators; Item #5: There was blinding of all subjects; Item #6: There was blinding of all therapists who administered the therapy; Item #7: There was blinding of all assessors who measured at least one key outcome; Item #8: Measures of at least one key outcome were obtained from more than 85% of the subjects initially allocated to groups; Item #9: All subjects for whom outcome measures were available received the treatment or control condition as allocated or, where this was not the case, data for at least one key outcome was analyzed by “intention to treat”; Item #10: The results of between- intervention group statistical comparisons are reported for at least one key outcome; Item #11: The study provides both point measures and measures of variability for at least one key outcome
