# Supplementary material for: Behavioral Treatment for Speech and Language in Primary Progressive Aphasia and Primary Progressive Apraxia of Speech: A Systematic Review
Source: Neuropsychol Rev. 2023 Oct 4;34(3):882–923. doi: 10.1007/s11065-023-09607-1 (PMC11473583; doi:10.1007/s11065-023-09607-1)
Supplement: Supplementary file 10 — Supplementary file10 (PDF 98.9 KB) [file 11065_2023_9607_MOESM10_ESM.pdf]

Wauters, L.D., Croot, K., Dial, H.R., Duffy, J.R., Grasso, S.M., Kim, E., Schaffer, K.M., Ballard, K.J., Clark, H.M., Kohley, L., Murray, L.L., Rogalski, E.J., Figeys, M., Milman, L., Henry, M.L., Behavioral treatment for speech and language in primary progressive aphasia and primary progressive apraxia of speech: A systematic review. *Neuropsychology Review.*

Corresponding author: Maya Henry, Department of Speech, Language, and Hearing Sciences, The University of Texas at Austin, 2504A Whitis Ave. (A1100), Austin, TX 78712-0114, E-mail: maya.henry@austin.utexas.edu.

Supplementary Materials 10: *Additional participant characteristics reported in the included studies* https://osf.io/ab8wq/?view_only=006f77b0c9414bb5a3595dc76bb455fd

Other sociodemographic characteristics

- Ethnicity/cultural identification
- Years of education, occupation, current employment status
- Current and/or past hobbies and interests
- Information about partner/children
- Residential setting, locality (urban, rural etc.), distance from treating clinic

Other clinical history characteristics

- Handedness
- Vision and hearing status
- Brain imaging results, including lateralization of atrophy
- Results of genetic screening
- Current, or previous history of, motor speech disorder, learning disorder, neurological disorder, depression, anxiety disorder, other psychiatric disorder
- Other medical co-morbidities, medications
- Personality characteristics
- Response to assessment, response to treatment
- Involvement of partner/adult children in care or treatment program
- Burden reported by individual with PPA or their partner

Other speech characteristics

- Overall fluency/dysfluency
- PPA severity
- Presence or absence of AOS and symptoms (articulatory groping, phonetic errors), dysarthria (and the type), motor speech disorder, stammering
- Slowing relative to premorbid speech rate
- Performance on the BDAE articulatory agility subtest
- Intelligibility level (related to the decision to introduce AAC)

Other language characteristics

- Whether the person was monolingual, bilingual, or multilingual, their first or native language (L1), proficiency in the language in which the study was conducted, proficiency in American Sign Language (ASL)
- Performance on aphasia and/or language batteries or their subtests (e.g. versions of AAT, BDAE, CADL, CAT, PALPA, WAB)
- Ratings on the PASS or language items in the CDR or FTLD-CDR Scales
- Statements about semantic knowledge, semantic association, single word comprehension (written and spoken word-picture-matching, nouns, verbs),

performance on semantic processing tests, e.g. Cambridge Semantic Battery subtests, the SYDBAT subtests, Pyramids and Palm Trees, Kissing and Dancing Test

- Self-reported word-finding difficulties, performance on written and/or spoken naming tests (e.g. BNT, GNT, NNB, Object and Action Naming Battery, Snodgrass and Vanderwart picture set)
- Performance on fluency tasks (phoneme, category, action, people)
- Typical utterance length, phonological errors or neologistic jargon in connected speech
- Information about single word repetition, sentence repetition, minimum number of syllables participants could repeat
- Information about reading aloud, surface dyslexia
- Information about writing, writing to dictation, spelling words and nonwords, surface dysgraphia
- History of developmental dyslexia or spelling difficulties
- Presence or absence of binary reversals
- Sentence comprehension, including comprehension of specific grammatical structures
- Information about agrammatism, sentences characterized by simple grammatical structures, ability to produce target verbs and/or nouns in a specified subject-verb-object (SVO) sentence, performance on grammatical tests e.g. NAT
- Supports used in functional communication (devices, other people)

Other cognitive and behavioral characteristics

- Statements about general level of cognitive ability, performance on cognitive screening tests (e.g. versions of the MMSE, MoCA, ACE), or level of non-verbal cognitive ability, IQ or estimated premorbid IQ
- Statements about independence in activities of daily living and level of support required if applicable, financial management, functioning at work and/or home and/or community, ability to maintain hobbies, use of computer technologies, general organization of home and appointment-keeping, driving
- Statements about whether the individual met criteria for a diagnosis of dementia, and criteria used, e.g. DSM-IV-TR
- Performance on cognitive/dementia assessment batteries (e.g. Battery of Lisbon, RBANS, CLQT), ratings on scales (e.g. FTLD-CDR)
- Statements about behavior, personality or emotional changes, including obsessive behavior
- Fatigue levels
- Statements about degree of impaired or preserved functioning in specific cognitive domains, with or without data on performance on individual tests:
    - Attention (selective attention, sustained attention, verbal attention, visual attention)
    - Visual and spatial skills
    - Memory (including short term memory, long term memory, episodic memory, semantic memory – see above, visual/visuospatial memory, verbal memory, recognition memory, immediate and delayed memory, learning). Tests include digit span forwards and back, spatial span forward and back, logical memory, paired associate learning
    - Object and face recognition (including famous faces, familiar faces, famous monuments, visual agnosia, finger agnosia)
    - Executive function (including disinhibition, impulsiveness, planning, problem solving, nonverbal reasoning, insight, cognitive flexibility). Tests include FAB, Stroop, Letter fluency, TMT, WCST

- Number processing and calculation
- Praxis (including ideomotor apraxia, apraxia for symbolic gestures)

Notes: AAC = augmentative and alternative communication, AAT = Aachener Aphasie Test (see studies included in review for details of tests as cited in the original studies), ACE = Addenbrooke's Cognitive Assessment, AOS = apraxia of speech, BDAE = Boston Diagnostic Aphasia Examination, BNT = Boston Naming Test, CADL = Communication Activities of Daily Living, CAT = Comprehensive Aphasia Test, CDR = Clinical Dementia Rating, CLQT = Cognitive-Linguistic Quick Test, DSM-IV-TR = Diagnostic and Statistical Manual of Mental Disorders Fourth Edition Text Revision, FAB = Frontal Assessment Battery, FTLD-CDR = Frontotemporal lobar degeneration-modified Clinical Dementia Rating, GNT = Graded Naming Test, MMSE = Mini-Mental State Examination, MoCA = Montreal Cognitive Assessment, NAT = Northwestern Anagram test, NNB = Northwestern Naming Battery, PALPA = Psycholinguistic Assessments of Language Processing in Aphasia, PASS = Progressive Aphasia Severity Scale, PPA = primary progressive aphasia, RBANS = *Repeatable Battery for the Assessment of Neuropsychological Status*, TMT = Trail Making Test, WAB = Western Aphasia Battery, WCST = Wisconsin Card-Sorting test.
