# Supplementary material for: Behavioral Treatment for Speech and Language in Primary Progressive Aphasia and Primary Progressive Apraxia of Speech: A Systematic Review
Source: Neuropsychol Rev. 2023 Oct 4;34(3):882–923. doi: 10.1007/s11065-023-09607-1 (PMC11473583; doi:10.1007/s11065-023-09607-1)
Supplement: Supplementary file 11 — Supplementary file11 (PDF 20 KB) [file 11065_2023_9607_MOESM11_ESM.pdf]

Wauters, L.D., Croot, K., Dial, H.R., Duffy, J.R., Grasso, S.M., Kim, E., Schaffer, K.M., Ballard, K.J., Clark, H.M., Kohley, L., Murray, L.L., Rogalski, E.J., Figeys, M., Milman, L., Henry, M.L., Behavioral treatment for speech and language in primary progressive aphasia and primary progressive apraxia of speech: A systematic review**.** *Neuropsychology Review*.

Corresponding author: Maya Henry, Department of Speech, Language, and Hearing Sciences, The University of Texas at Austin, 2504A Whitis Ave. (A1100), Austin, TX 78712-0114, E-mail: maya.henry@austin.utexas.edu.

Supplementary Materials 11: *Additional study design characteristics extracted during the review* https://osf.io/ab8wq/?view_only=006f77b0c9414bb5a3595dc76bb455fd

**Study design characteristic**	**Number of studies /103 (%)**
**Reporting quality**	
• Study procedures replicable as reported	63 (61.2%)
**Internal validity**	
• Reliability of outcome variables is reported	22 (21.3%)
**External validity**	
• Replication of results within study	35 (34.0%)
